# LncRNA5251 inhibits spermatogenesis via modification of cell-cell junctions

**DOI:** 10.1186/s13062-023-00381-x

**Published:** 2023-06-15

**Authors:** Cong Zhang, Dongxin Lu, Tong Niu, Zhongyi Sun, Yandi Wang, Xiao Han, Bohui Xiong, Wei Shen, Qingyuan Sun, Yong Zhao, Weidong Zhang, Yanni Feng

**Affiliations:** 1 Laboratory of Animal Reproductive Physiology and Disease, College of Veterinary Medicine, Qingdao Agricultural University, Qingdao, 266109 P. R. China; 2 Urology Department, Shenzhen University general hospital, Shenzhen, 518055 P. R. China; 3 College of Life Sciences, Qingdao Agricultural University, Qingdao, 266109 P. R. China; 4 Fertility Preservation Lab, Reproductive Medicine Center, Guangdong Second Provincial General Hospital, Guangzhou, 510317 P. R. China; 5 College of Science, Health, Engineering and Education, Murdoch University, Perth, 6150 Australia

**Keywords:** Male fertility, lncRNA5251, Spermatogenesis, Cell junctions

## Abstract

**Background:**

Male factors-caused decline in total fertility has raised significant concern worldwide. LncRNAs have been identified to play various roles in biological systems, including spermatogenesis. This study aimed to explore the role of lncRNA5251 in mouse spermatogenesis.

**Methods:**

The expression of lncRNA5251 was modulated in mouse testes in vivo or spermatogonial stem cells (C18-4 cells) in vitro by shRNA.

**Results:**

The sperm motility in two generations mice after modulation of lncRNA5251 (muF0 and muF1) was decreased significantly after overexpression of lncRNA5251. GO enrichment analysis found that knockdown lncRNA5251 increased the expression of genes related to cell junctions, and genes important for spermatogenesis in mouse testes. Meanwhile, overexpressing lncRNA5251 decreased the gene and/or protein expression of important genes for spermatogenesis and immune pathways in mouse testes. In vitro, knockdown lncRNA5251 increased the expression of genes for cell junction, and the protein levels of some cell junction proteins such as CX37, OCLN, JAM1, VCAM1 and CADM2 in C18-4 cells. LncRNA5251 is involved in spermatogenesis by modulation of cell junctions.

**Conclusion:**

This will provide a theoretical basis for improving male reproductive ability via lncRNA.

**Supplementary Information:**

The online version contains supplementary material available at 10.1186/s13062-023-00381-x.

## Introduction

At present, the decline of male fertility has caused widespread concern. Reasons for reduced reproductive ability and even infertility include obesity, diabetes, environmental chemicals and genetic factors [1–4]. Many studies have reported that air pollutants are key factors affecting human reproductive health [5, 6]. Spermatogenesis is the process to produce male gametes, which is a fine-regulated process in which germ cells produce mature sperm through a series of proliferation and differentiation [7]. During the transformation of spermatogonia into sperm, various types of germ cells migrate from the basal to the seminiferous lumen, and the interaction between germ cells, Sertoli cells, as well as germ cells and Sertoli cells playing vital roles for this process. The blood-testis-barrier (BTB) is formed by special connections between Sertoli cells, including tight junctions (TJ), cytoplasmic specialization, desmosomes, and gap junctions (GP). The connections between cells communicate with each other through secreted factors and signal molecules, forming a bidirectional signaling system to promote spermatogenesis. The complex formed between junction proteins enables BTB to be opened and closed in an orderly manner, which promotes the migration of developing germ cells from the basement membrane to the lumen without damaging the integrity of BTB [8].Therefore, the cell junction proteins Cx43, Occludin and Claudin play an important roles in BTB. It is suggested that BTB is one of the tightest tissue barriers in mammals [9–11], and it provides a functional microenvironment for spermatogenesis.

LncRNA is a type of RNA with a length more than 200 nt and no coding capacity, which is usually transcribed by RNA polymerase II/III [12]. It is rich in species, complex in function, low in sequence conservation among different species, specific in tissue and developmental stages [13]. The lncRNA sequence is poorly conserved, however it is an important regulator of gene expression, which can play crucial roles in different biological processes [14, 15]. LncRNA can regulate the expression of target genes at transcriptional, post-transcription and epigenetic levels [16]. It has been noted that lncRNA is involved in spermatogenesis, which plays an important regulatory role in spermatogonial stem cell proliferation [17], germ cell meiosis [18] and sperm maturation [19]. A small number of lncRNAs such as Tsx, Drm, etc. have been verified and functionally characterized, but the functions of most lncRNAs in spermatogenesis are still poorly understood.

Our previous investigation uncovered that air pollutants (NH_3_, H_2_S)-decreased mouse male fertility can be heritable, and many lncRNAs were altered in mouse sperm. However, we did not know the functions of these changed lncRNAs in mouse sperm. The aim of this study was designed to explore the functions of the altered mouse sperm lncRNAs and the underlying mechanisms, which will provide a theoretical basis for improving male reproductive ability at molecular level, and provide a scientific guidance for improving male reproductive ability.

## Materials and methods

**Study design.** All animal procedures used in this study were approved by the Animal Care and Use Committee of the Institute of Animal Sciences of Chinese Academy of Agricultural Sciences. Mice were maintained in specific pathogen-free (SPF) environment under a light: dark cycle of 12:12 h, at a temperature of 23 ℃ and humidity of 50–70%; they had free access to food (chow diet) and water [20–22].

**Fig. 1:**
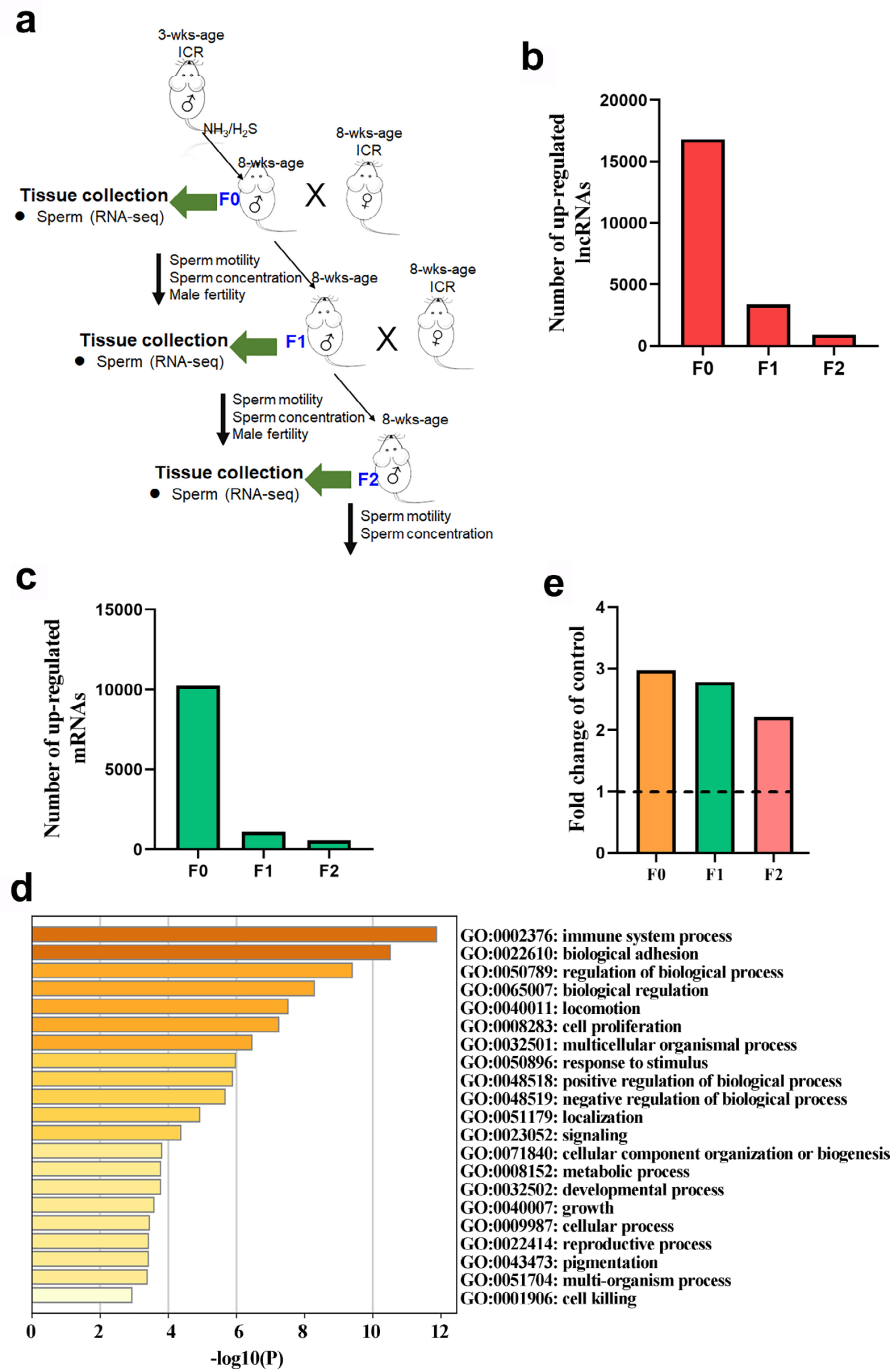
**LncRNA and mRNA expression in three generations mouse testis samples by RNA-seq analysis.** (**a**) Study scheme. Gene changes in sperm of three generations of mice treated with NH_3_/H_2_S. (**b**) The number of known up-regulated lncRNAs in the sperm of three generations of mice after NH_3_/H_2_S treatment. (**c**) The number of known increased mRNAs in the sperm of three generations of mice after NH_3_/H_2_S treatment. (**d**) GO enrichment of target genes that known up-regulated lncRNAs in F0 generation. (**e**) The expression of lncRNA5251 in the sperm of three generations of mice

*Animal Experiment I: NH*_*3*_*/H*_*2*_*S study* [20, 22]. Three-week-old ICR male mice were dosed with phosphate buffered saline (PBS) as vehicle control (Control group) or with Na_2_S-50 mg/kg body weight (BW) + NH_4_Cl-50 mg/kg BW (NH_3_/H_2_S group) [23, 24] once daily for 5 weeks. There were 60 mice/group. The volume of gavage was 0.1 ml/mouse/day. Subsequently, 30 mice/treatment were humanely terminated for the analysis of sperm quality and other parameters. A further 30 mice/treatment were mated with normal (untreated) ICR female mice (male: female; 1:2). After birth of the F1 litter, the number of live pups/litter was counted and all mice were raised similarly without further treatment. At the age of 8 weeks (F1), 30 male mice/treatment were humanely terminated for analysis of sperm quality and other parameters. A further 30 male mice/treatment were mated with normal ICR female mice (male: female; 1:2) and subsequently underwent a similar procedure. After birth of the F2 litter, the number of live pups/litter was counted and all mice were raised in a similar manner without further treatment (Study scheme in Fig. 1a).

**Fig. 2:**
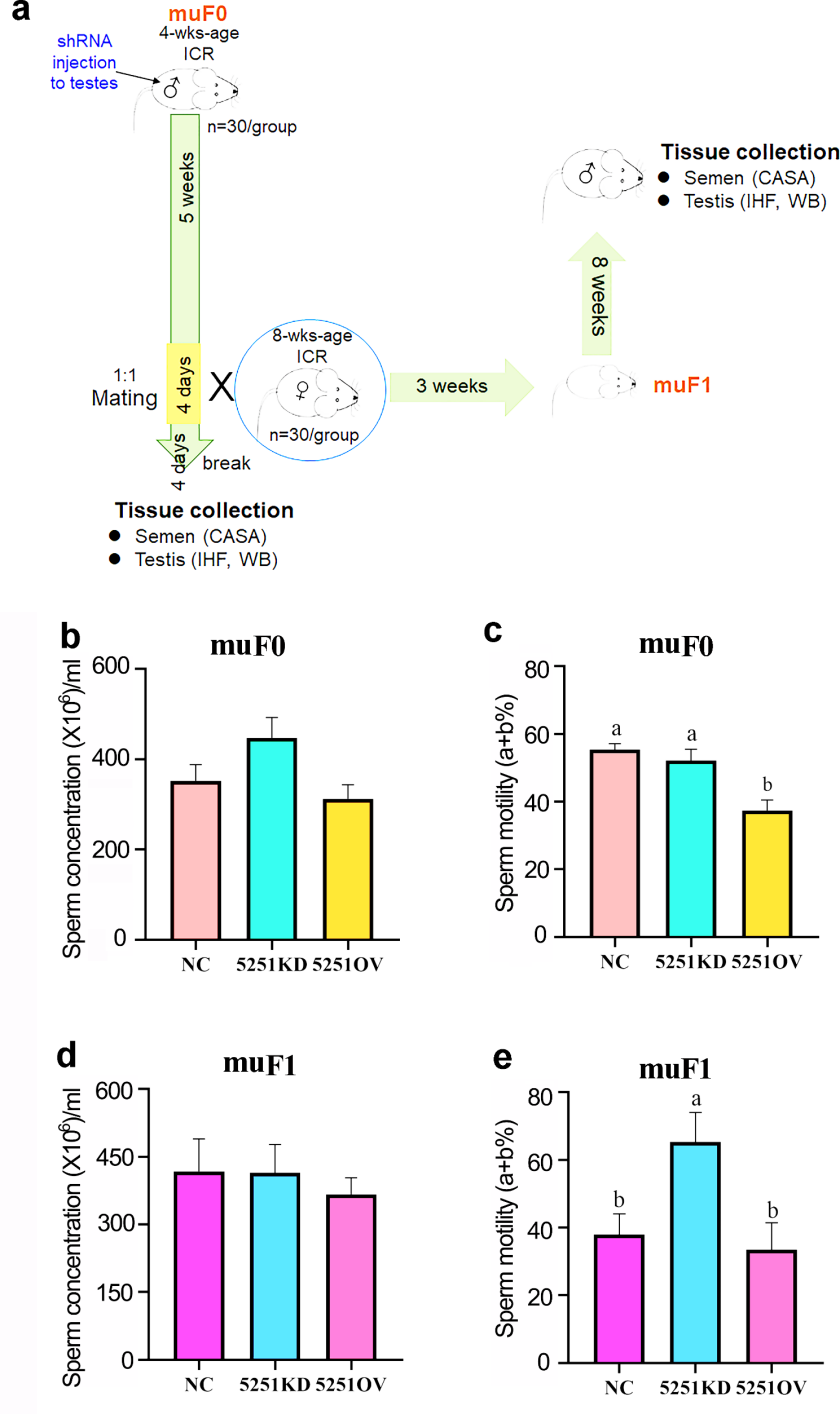
**Overexpression of lncRNA5251 decreased mouse semen quality.** (**a**) Study scheme. Sperm quality of mice after shRNA treatment. (**b**) Sperm concentration of F0 mice. (**c**) Sperm motility of F0 mice. (**d**) Sperm concentration of F1 mice. (**e**) Sperm motility of F1 mice. Data present as Average ± SEM. a, b indicate a significant difference among different treatments (p < 0.05)

*Animal Experiment II: Knockdown or overexpression of lncRNA5251 in mouse testes* [25, 26]. The procedure for the production of shRNA and In vivo virus grafting have been published in our recent article [25]. (1) *Production of lentivirus.* Lentivirus production was performed as described previously [25, 26]. Knockdown Lenti-lncRNA5251 (5251KD) was cloned using the lentivirus-*3* (LV3; 3NC) vector as a backbone (Fig. S1a), while overexpression of lenti-lncRNA5251 (5251OV) was cloned using the lntivirus-5 (LV5) vector as a backbone (Fig. S1b). There were three knockdown shRNAs at three different positions for lncRNA5251, and the sequences for each position and NC are listed in Table S1. The full length of lncRNA5251 (Table S2) was inserted into LV5 to make the overexpression lentivirus (5NC was the LV5 vector). The efficiency and specificity of shRNA knockdown were determined by transfecting into 293T cells using Lipofectamine 2000 (Invitrogen, Waltham, MA, USA; #11668-027), followed by analysis at 60 h post-transfection by qPCR. Lentivirus production was then performed as shown in Fig. S1c. Approximately 10^9^ infectious viral particles/ml were obtained. (2) In vivo virus grafting and sample collection. In vivo virus grafting was performed as previously described [25, 26]. In current investigation, four-week-old ICR male mice were used. Briefly, four-week-old ICR male mice were anesthetized with isofluorane. Microinjections were performed using 26-gauge needles connected to a 100 µL syringe. Virus (3 µl with titer greater than 3 × 10^8^/ml) for each position for knockdown shRNA [in total 9 µl with a titer > 6 × 10^8^/ml for 3NC, or lncRNA5251 (KD) individually] were mixed and then injected into the testes. For overexpression, virus (9 µl with titer greater than 6 × 10^8^/ml) for 5NC, or lncRNA5251 (OV) individually were mixed and then injected into the testes. Then the mice were raised regularly for five weeks till nine weeks of age (Fig. 2a; muF0). The mice mated with normal 8-week-age ICR female mice (male: female, 1:1) for four days. The male mice were kept for another four days, then terminated for collection samples and analysis. The female mice were maintained regularly till the delivery of offspring. The offspring was raised regularly till eight weeks age. Then the male offspring (muF1) were terminated for collection of samples and analysis (Fig. 2a).

*Cell culture experiment*: *Knockdown or overexpression of lncRNA5251 in mouse spermatogonia cell line C18-4 cells* [25]. The C18-4 cell line (mouse spermatogonia stem cells; Donated by Dr. Wenxian Zeng, Northwest A&F University) was held in DMEM/F12 (Gibco) supplemented with 10% (FBS), 2 mM L-glutamine (Invitrogen), and 100 U/ml penicillin and streptomycin (Invitrogen) [25, 27, 28]. The cells were transfected with shRNA in 6-well plates. For knockdown, tthree respective shRNAs for each position were mixed together (titer > 3 × 10^8^/ml) with RNAi-mate for the transfection of C18-4, while for overexpression, shRNAs (titer > 3 × 10^8^/ml) with RNAi-mate for the transfection of C18-4. The transfection medium was changed after 12 h. Stable transfected cells were cultured in a similar manner to the non-transfected cells in their respective media.

**Evaluation of spermatozoa motility using a computer-assisted sperm analysis system.** Spermatozoa motility was assessed using a computer-assisted sperm assay (CASA) method according to World Health Organization guidelines and reported in our early studies [20, 21, 29–31].

***Morphological observations of spermatozoa.****T*he extracted murine caudal epididymis were placed in RPMI medium, finely chopped, and then Eosin Y (1%) was added for staining as described previously [20, 21, 31].

**Assessment of acrosome integrity.** The procedure of analysis of acrosome integrity was reported in our recent articles [20, 21, 31].

**RNA Isolation and RNA-seq analyses**. The RNA-seq analysis procedure has been published in our recent article [31].

**Histopathological analysis.** Testicular tissues were fixed in 10% neutral buffered formalin, paraffin embedded, cut into 5 μm sections and subsequently stained with hematoxylin and eosin (H&E) for histopathological analysis [20, 21, 31].

**Western blotting.** Western blotting analysis of proteins was carried out as previously reported [20, 21, 31]. The information for primary antibodies (Abs) were listed in Table S3.

**Detection of protein levels and location in testis using immunofluorescence staining.** The methodology for immunofluorescence staining of testicular samples is reported in our recent publications [20, 21, 31]. Table S3 listed the primary antibodies used in this study.

***Immunofluorescence staining with frozen sections for C18-4 cells***. The protocol for immunofluorescence staining analysis of C18-4 cells was reported in our recent article [32].

**Statistical analysis.** Data were analyzed using SPSS statistical software (IBM Co., NY) with one-way analysis of variance (ANOVA) followed by LSD multiple comparison tests or T-test. The data were shown as the mean ± SEM. Statistical significance was based on p < 0.05.

## Results

### LncRNA5251 was increased in F0, F1 and F2 mouse sperm after NH_3_/H_2_S treatment transgenerationally

It has been reported in our previous articles that sperm quality (concentration and motility) and male fertility were decreased by the treatment of NH_3_ + H_2_S [20, 22]. After treating with NH_3_ and H_2_S, the number of known up-regulated lncRNAs in the mouse sperm of F0, F1, and F2 generations showed a downward trend in Fig. 1b. Similarly, mRNA-seq results showed that the number of known up-regulated mRNAs in the mouse sperm of three generations were 10,242, 1113, and 563 respectively which also decreased sequentially (Fig. 1c). Therefore, we are very interested in these up-regulated lncRNAs. The predicted target genes of up-regulated lnRNAs in F0 generation mice were determined by GO enrichment analysis to search for the functions. It was interesting to notice that biological adhesion, cell proliferation, development process, and reproduction process terms were enriched in the comparison of treatment group (NH_3_ + H_2_S) vs. control group (Fig. 1d). Sixty-five known lncRNAs were up-regulated in common in the sperm of three generations of mice. It was interesting to notice that the fold changes of lncRNA5251 were great than 2 folds in the three generations samples (Fig. 1e). We would like to explore the role of it in spermatogenesis and the underlying mechanisms.

### Changs in lncRNA5251 expression altered sperm concentration and motility in mice in vivo

In order to explore the effect of lncRNA5251 on the reproduction of male mice, lncRNA5251 was overexpressed (5251OV) and inhibited (5251KD) in the testis of F0 mice by shRNA (muF0). The sperm concentration in muF0 generation mice was increased by 5251KD and decreased by 5251OV, although it was not significant (Fig. 2b). However, the sperm motility was significantly decreased by 5251OV while it was no change in 5251KD group (Fig. 2c). The sperm concentration was reduced in muF1 5251OV group even though it was not significantly (Fig. 2d). However, the sperm motility was increased in muF1 5251KD group (Fig. 2e). These results indicated that lncRNA5251 was involved in the spermatogenesis to regulate sperm concentration or sperm motility. And overexpression of lncRNA (5251OV) had the negative effects on sperm quality while knockdown of lncRNA5251 increased sperm quality. The results matched the lncRNA sequencing data that lncRNA5251 was increased in the sperm while mouse sperm concentration and motility were diminished [20, 22].

### Modification of lncRNA5251 expression affected spermatogenesis in muF0 mice

**Fig. 3:**
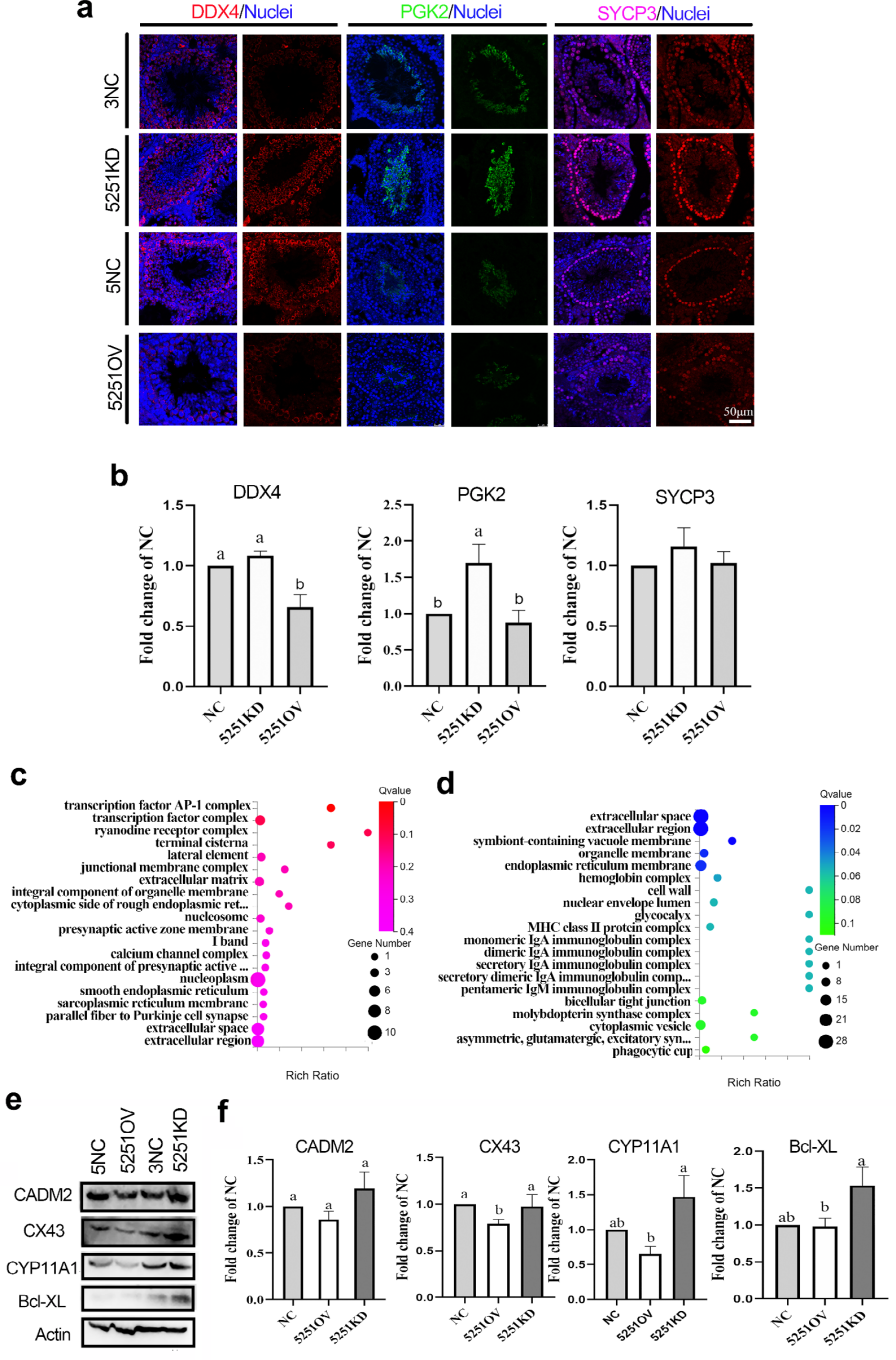
**Overexpression of lncRNA5251 disrupted spermatogenesis in F0 mice.** (**a**) The protein staining of DDX4, PGK2 and SYCP3 in F0 mice testis after shRNA treatment detected by IHF. (**b**) The quantitative data for IHF in (**a**). (**c**) Gene ontology (GO) enrichment of up-regulated genes after knockdown of lncRNA5251. (**d**) GO enrichment of decreased genes after overexpression of lncRNA5251. (**e**) The protein levels of CADM2, CX43, CYP11A1 and BCL-XL detected by WB in F0 mouse testes. (**f**) The quantitative data for WB in (**e**). Data present as Average ± SEM. a, b indicate a significant difference among different treatments (p < 0.05)

Since modification of lncRNA5251 expression affected sperm concentration and motility, we set out to explore the effects of modification of lncRNA5251 expression on spermatogenesis. The important proteins for spermatogenesis were determined in mouse testis samples by immunofluorescence staining (IHF). The results showed that the expression of germ cell marker DDX4 was significantly reduced in 5251OV group, sperm protein maker PGK2 was significantly increased in muF0 5251KD group, while the meiosis marker SYCP3 was not change significantly (Fig. 3a and b). The data indicated that modification the expression of lncRNA5251 impacted on the spermatogenesis to affect sperm quality. At the same time, the gene expression in the muF0 mouse testis samples showed that modification the expression of lncRNA5251 altered the gene expression. Compared to control (NC), there were 57 increased genes while 86 decreased genes in muF0 5251KD. There were 55 upregulated and 134 gene downregulated in muF0 5251OV compared to NC (Fig. S2). GO enrichment analysis showed that the up-regulated genes were enriched in junction membrane complex and extracellular region in 5251KD group (compared to NC) (Fig. 3c). It was interesting that down-regulated genes in the 5251OV group (compared to NC) were also enriched in organelle membranes, bicellular tight junction and multiple immunoglobulin complexes (Fig. 3d). At the same time, the protein levels of the cell junction proteins CADM2 and CX43 were decreased in the 5251OV group (compared to NC; Fig. 3e and f). Moreover, the protein levels of steroid hormone production protein CYP11A1 and apoptosis protein Bcl-xl were increased in 5251KD group (compared to NC) (Fig. 3e and f). It has been reported that the cell junction complexes are the key regulators for the interaction between cell junctions, which can regulate the “opening and closing” of BTB and play a vital role in spermatogenesis [33]. The data suggested that lncRNA5251 is involved in the spermatogenesis by modulating the formation of cell junctions, and the steroid hormone production.

### Alteration of lncRNA5251 expression even impacted on spermatogenesis in muF1 mice

**Fig. 4:**
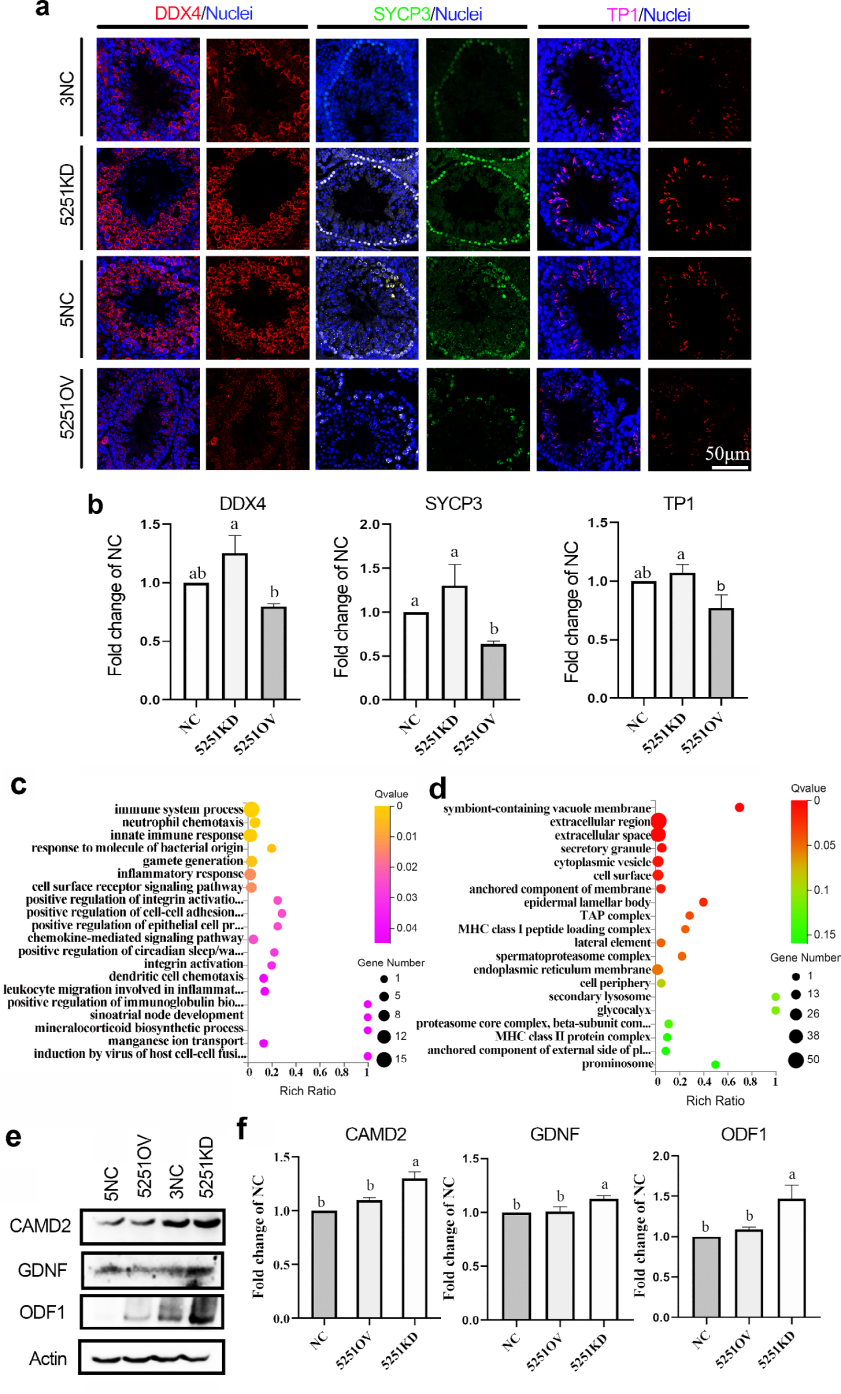
**Spermatogenesis was upset in F1 mice after overexpression lncRNA5251.** (**a**) The protein staining of DDX4, SYCP3, TP1 and PGK2 in F1 mice testis after shRNA treatment by IHF. (**b**) The quantitative data for IHF in (**a**). (**c**) GO enrichment analysis of up-regulated genes after inhibiting lncRNA5251. (**d**) GO enrichment analysis of down-regulated genes after overexpressing lncRNA5251. (**e**) The protein levels of CADM2, GDNF and ODF1 detected by WB in F1 mouse testes. (**f**) The quantitative data for WB in (**e**). Data present as Average ± SEM. a, b indicate a significant difference among different treatments (p < 0.05)

Modification of the expression of lncRNA5251 altered the spermatogenesis in muF1 mouse testis. The IHF data showed that the expression levels of germ cell marker DDX4, meiosis marker SYCP3, and transit protein TP1 were significantly reduced in 5251OV group F1 mouse testis samples (compared to NC; Fig. 4a and b). At the same time, the gene expression data showed that, compared to control (NC), there were 104 increased genes while 222 decreased genes in 5251KD. There were 152 upregulated and 214 gene downregulated in 5251OV compared to NC (Fig. S3). GO enrichment analysis showed that the up-regulated genes were enriched in biological processes related to spermatogenesis, such as gamete generation, positive regulation of cell-cell adhesion, and positive regulation of epithelial cell proliferation in 5251KD group (compared to NC) (Fig. 4c). It was interesting that down-regulated genes in the 5251OV group (compared to NC) were enriched in the cell surface, anchored component of membrane, and spermatoproteasome complex (Fig. 4d). At the same time, the protein levels of the cell junction proteins CADM2 were increased in the 5251KD group (compared to NC; Fig. 4e and f). Moreover, the protein levels of important proteins for spermatogenesis or sperm quality GDNF and ODF1 were increased in 5251KD group (compared to NC) (Fig. 4e and f). The data suggested that lncRNA5251 is not only involved in the spermatogenesis muF0 mouse, but also in muF1 mouse which indicated that lncRNA5251 is very important in spermatogenesis.

### Inhibition of lncRNA5251 increased cell junction proteins in C18-4 cells

**Fig. 5:**
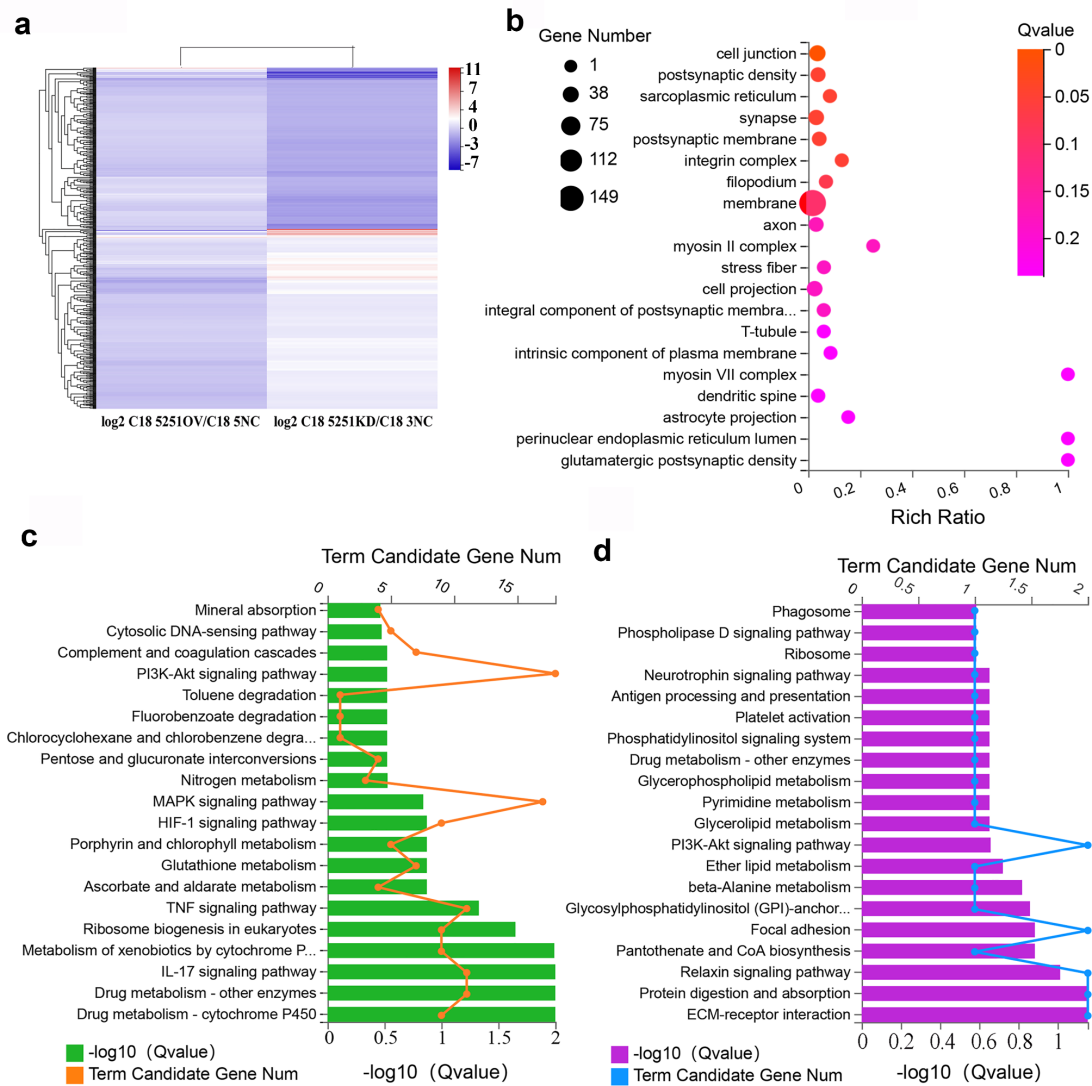
**Gene expression in C18-4 cells after modification of lncRNA5251 expression.** (**a**) Gene expression heatmap of C18-4 cells after modification of lncRNA5251 expression. (**b**) GO enrichment analysis of the increased genes in C18-4 cells after inhibiting lncRNA5251 expression. (**c**) KEGG enrichment of the genes after inhibition of lncRNA5251 in C18-4 cells. (**d**) KEGG enrichment analysis of the genes in C18-4 cells after overexpression of lncRNA5251.

**Fig. 6:**
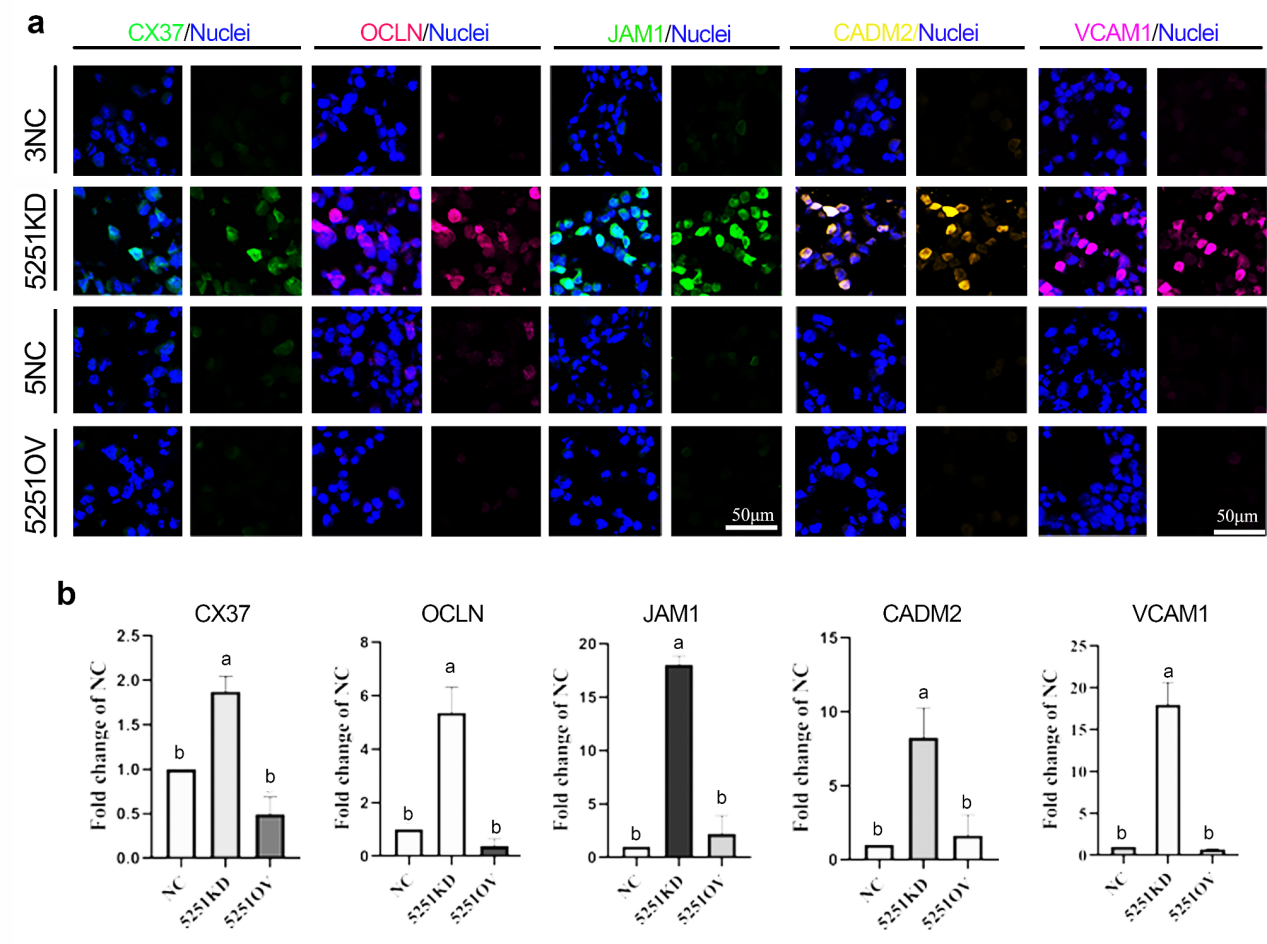
**Expression of cell junction related protein after modification lncRNA5251.** (**a**) The protein staining of CX37, OCLN, JAM1, CADM2 and VCAM1 in C18-4 cells after inhibition and overexpression of lncRNA5251detected by IHF. (**b**) The quantitative data for IHF in (**a**). Data present as Average ± SEM. a, b indicate a significant difference among different treatments (p < 0.05)

Mature sperm is derived from spermatogonial stem cell through a series of divisions and differentiations. C18-4 cell line is a commonly used spermatogonial stem cell model in vitro. Since lncRNA5251 was found in three generational mouse sperm, C18-4 cells were applied to explore the deep mechanisms of lncRNA5251 impact on spermatogenesis and sperm quality. The expression of lncRNA5251 in C18-4 cells was inhibited (C18-5251KD) and overexpressed (C18-5251OV) by shRNA. The gene expression after modification of the expression of lncRNA5251 was determined by RNA-seq analysis. The heatmap showed that 321 genes were up-regulated while 288 genes were down-regulated in the C18-5251KD group (compared to NC), however, 17 genes were up-regulated and 25 genes were down-regulated in the C18-5251OV group (compared to NC; Fig. 5a). GO enrichment analysis showed that the genes up-regulated in the KD group were enriched in cell junction, integrin complex, myosin II complex, T-tubules and others related to cell adhesion and junction (Fig. 5b). Subsequently, KEGG analysis showed that the changed genes were enriched in PI3K-AKT and MAPK signaling pathways in C18-5251KD group (compared to NC; Fig. 5c), while the changed genes of in C18-5251OV group were enriched in the pathways ECM receptor interaction, focal adhesion, and PI3K-AKT pathway (Fig. 5d). These data indicated that lncRNA5251 is involved in the cell adhesion and junction formation in mouse testis. Then the protein levels of cell junction and adhesion proteins were verified by IHF analysis which included CX37, CX43, ZO-1, Occludin (OCLN), JAM1, VCAM1, Desmoglein 2 (DSG2), Catenin, Claudin 11, E-cadherin and CADM2. The data showed that the expression levels of five cellular adhesion/junction proteins CX37, Occludin (OCLN), JAM1, VCAM1 and CADM2 in C18-4 cells were significantly increased in 5251KD group compared to NC (Fig. 6a and b), while other the data for other proteins were not significant (Data not shown). The data from C18-4 cells further suggested that lncRNA5251 is involved in cell-cell junctions to modulate spermatogenesis.

## Discussion

LncRNA lacks an open reading frame and was regarded as a by-product in the transcription process in the early time, so it has not received much attention. With the advancement of sequencing technology, lncRNA has truly entered people’s field of vision. This non-protein coding RNA, once regarded as “junk”, plays important roles in many biological processes such as embryonic development, cell differentiation and tumor metastasis [14, 15, 34]. Studies have shown that lncRNA is also involved in spermatogenesis [35]. Many lncRNAs have different expression levels and tissue specificities at different stages of mammalian spermatogenesis, and they are involved in the regulation of gene silencing, cell division, gonadal development, and sex determination [36]. For example, Mrhl mediates the meiosis process in mouse testis by regulating the expression of Sox8 [37]. LncRNA033862 can regulate the self-renewal of spermatogonia stem cell (SSC) by acting as a transcriptional activator of Gfra1 [38]. Recently, the decline of male fertility caused by environmental pollutions has attracted more and more attention. As previously reported, the reduction in sperm quality after NH_3_ and H_2_S treatment was transgenerational [20, 22]. In current investigation, we found many lncRNAs were altered after NH_3_ + H_2_S treatment in three generational mouse sperm (F0, F1, F2). And lncRNA5251 was interesting because it was increased in the three generational mouse sperm. And we explored the effects and underlying mechanisms of lncRNA5251 on sperm quality (concentration and motility) and spermatogenesis by knocking down or overexpressing the expression of lncRNA5251 through shRNA. Interestingly, the results from in vivo experiments showed that the sperm quality (concentration and motility) of F0 and F1 mice was decreased after overexpression of lncRNA5251 which suggested that it is involved in spermatogenesis.

Spermatogenesis is a complex process including mitotic cell division, meiosis and the process of spermiogenesis [39]. RNA-seq of testis samples showed that the differentially expressed genes in 5251KD group (compared to NC) or in 5251OV group (compared to NC) were enriched in the immune, spermatogenesis process, especially in the pathways of cell junctions, regardless of the muF0 or muF1 generation. And germ cell marker, meiosis and sperm proteins, such as DDX4, SYCP3, PGK2 and TP1 were altered in 52521KD or 5251OV group which further suggested lncRNA5251 regulates spermatogenesis. Moreover, the expression of the cell connexins CADM2 and CX43 was diminished in 5251OV group by WB analysis which further confirmed the RNA-seq data.

In the process of spermatogenesis, germ cells migrate from the base to the lumen through BTB, completing the process of sperm formation under various adjustments [40]. And BTB works through cell junctions which are composed of a variety of proteins. For example, the tight junction (TJ) proteins Occludin and Claudin, gap junction proteins CX43 and CX37 [41] as well as adhesion junction (AJ) protein JAMs [42] are important for the barrier integrity of epithelial tissues [43]. TJ and AJ can be connected by adaptors (such as ZO-1), which are structurally involved in enhancing BTB [11]. Totally, cell junction proteins work together to maintain the homeostasis of the testicular environment to promote spermatogenesis. In C18-4 cells, the expression levels of cell connexin CX37, OCLN, JAM1, VCAM1 and CADM2, were significantly increased in 5251KD group. In vitro experiments further confirmed that lncRNA5251 is involved in spermatogenesis through cell junctions.

## Conclusion

In summary, the in vivo and in vitro results together prove that lncRNA5251 regulates spermatogenesis via cell-cell junctions. This work provides a theoretical basis for improving male reproductive ability through lncRNA to help the infertile patients to be parenthood.

## Electronic supplementary material

Below is the link to the electronic supplementary material.

Supplementary Material 1

Supplementary Material 2

Supplementary Material 3

Supplementary Material 4

Supplementary Material 5

Supplementary Material 6

Supplementary Material 7

## Data Availability

Sperm, testes and C18-4 cells RNA-seq raw data were deposited in NCBI’s Gene Expression Omnibus under accession number GSE137630 (sperm), GSE142724 (muF0, testes), GSE142725 (muF1, testes) and GSE142726 (C18-4 cells), respectively.

## Abbreviations

Abs: Antibodies
AJ: Adhesion junction
ANOVA: Analysis of variance
BTB: Blood-testis-barrier
CADM2: Cell Adhesion Molecule 2
CASA: Computer-assisted sperm assay
CX37: Connexin 37
Cx43: Connexin 43
DDX4: DEAD-Box Helicase 4
ECM: Extracellular matrix
GDNF: Glial Cell Derived Neurotrophic Factor
GO: Gene Ontology
GP: Gap junction
H&E: Hematoxylin and eosin staining
IHF: Immunofluorescence staining
JAM1: Junctional Adhesion Molecule1
KD: Knockdown
KEGG: Kyoto Encyclopedia of Genes and Genomes
NC: Control
OCLN: Occludin
ODF1: Outer Dense Fiber Of Sperm Tails 1
OV: Overexpression
PGK2: Phosphoglycerate Kinase 2
SPF: Specific pathogen-free
SYCP3: Synaptonemal Complex Protein 3
SSC: Spermatogonia stem cell
TJ: Tight junction
VCAM1: Vascular Cell Adhesion Molecule 1
WB: Western blotting
ZO-1: Tight Junction Protein 1.
