# Supplementary figures and images for: LncRNA5251 inhibits spermatogenesis via modification of cell-cell junctions
Source: Biol Direct. 2023 Jun 15;18:31. doi: 10.1186/s13062-023-00381-x (PMC10268499; doi:10.1186/s13062-023-00381-x)

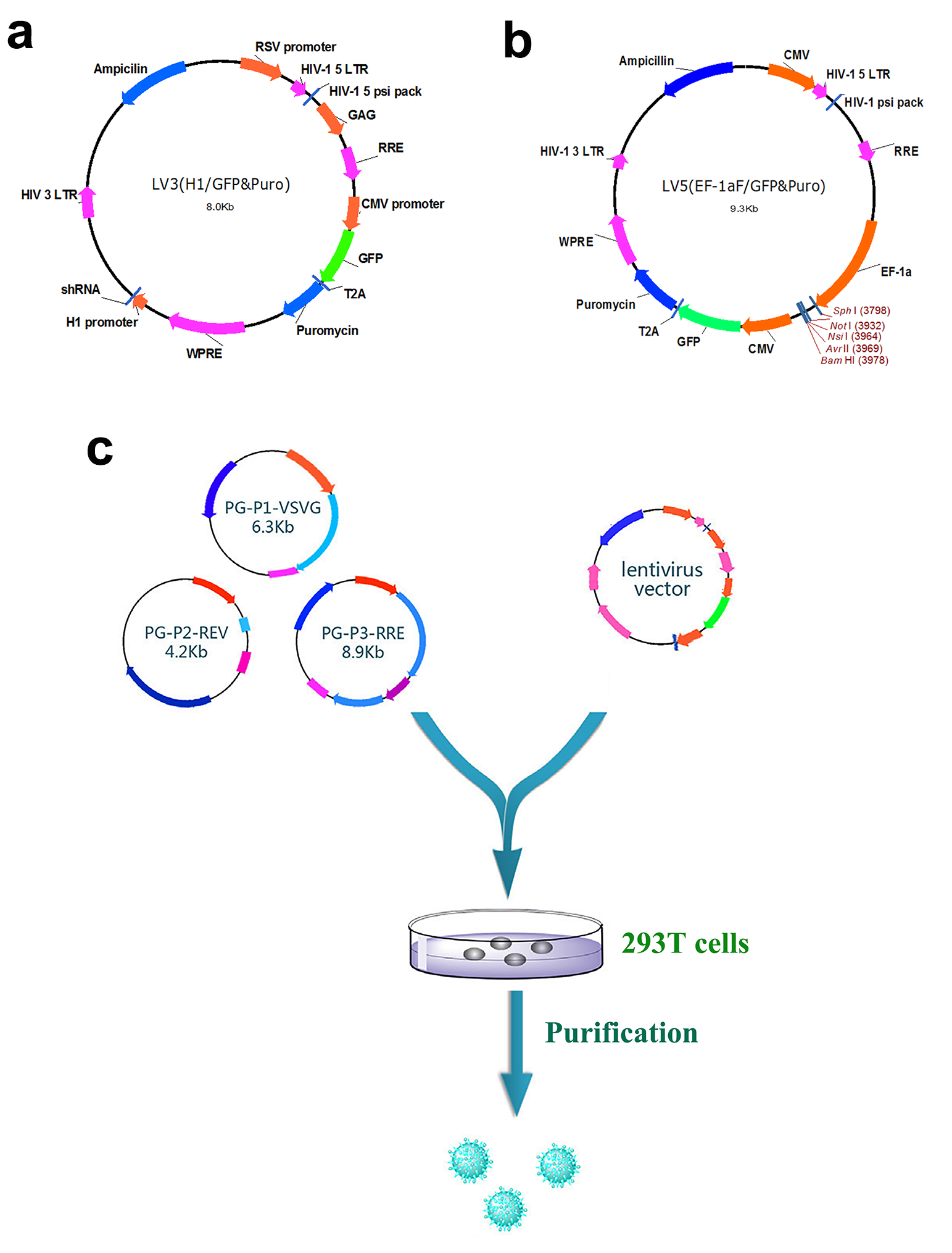

Supplement: Supplementary file 1 — Supplementary Material 1 [file 13062_2023_381_MOESM1_ESM.tif]

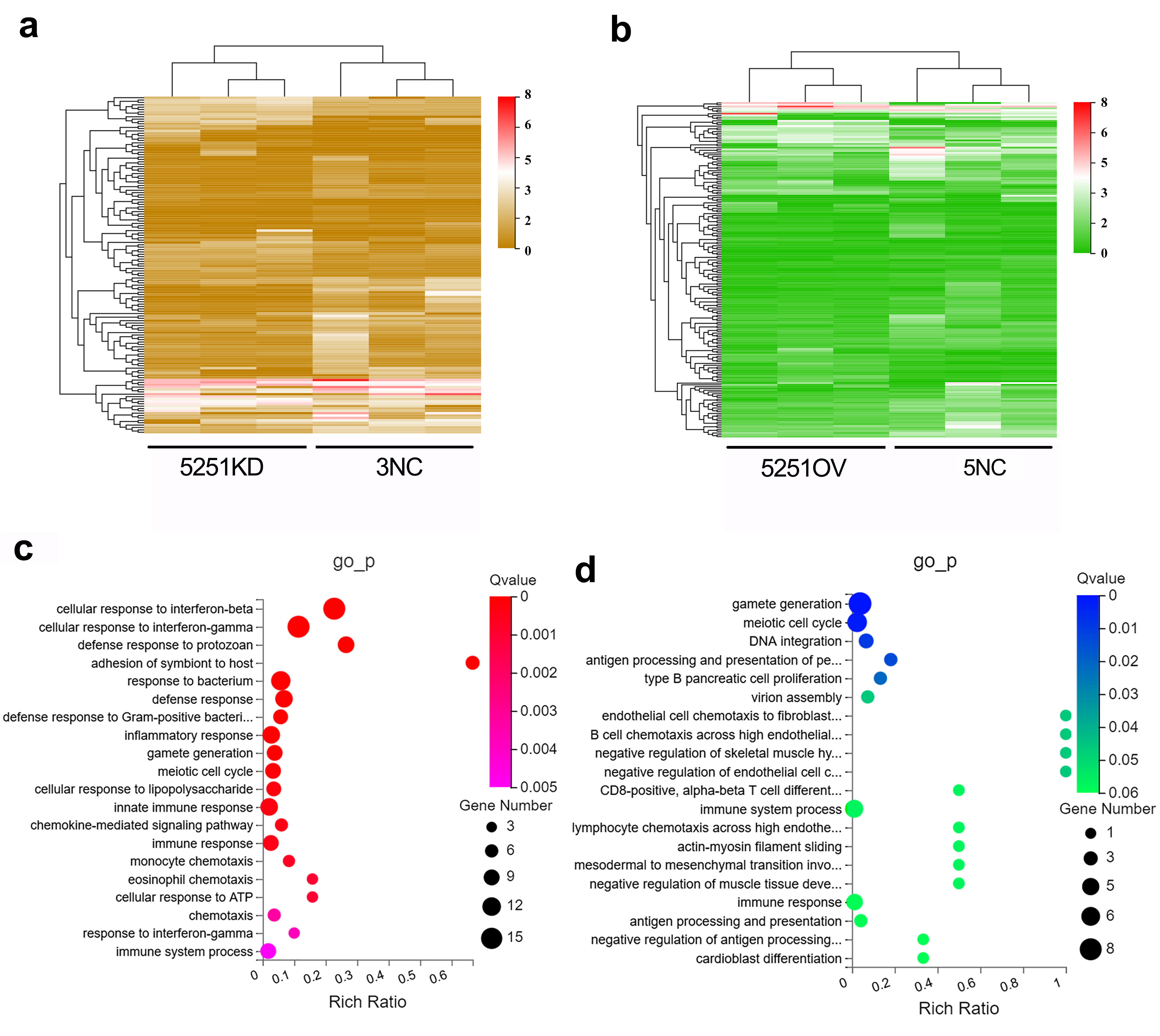

Supplement: Supplementary file 2 — Supplementary Material 2 [file 13062_2023_381_MOESM2_ESM.tif]

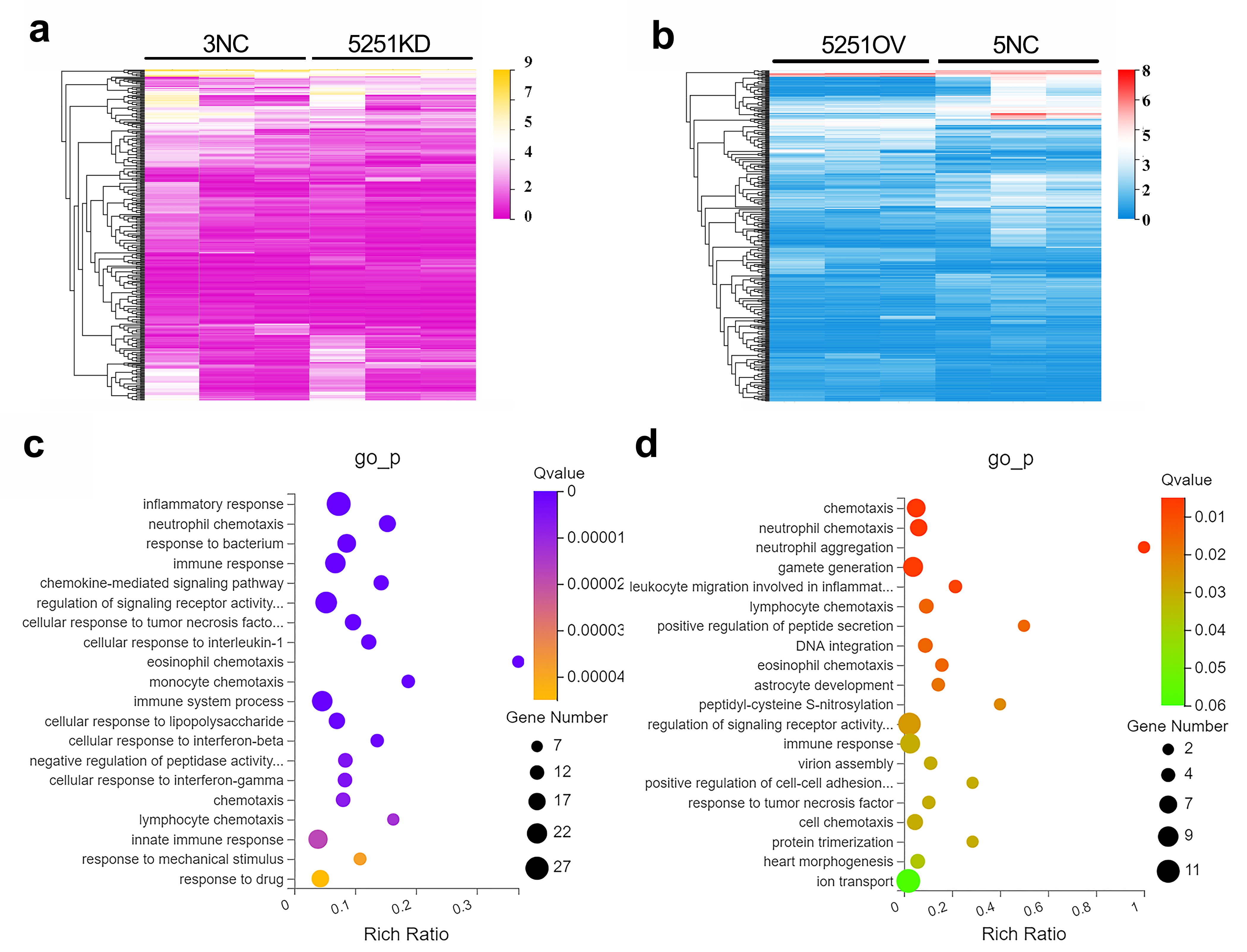

Supplement: Supplementary file 3 — Supplementary Material 3 [file 13062_2023_381_MOESM3_ESM.tif]
