# Supplementary material for: LncRNA5251 inhibits spermatogenesis via modification of cell-cell junctions
Source: Biol Direct. 2023 Jun 15;18:31. doi: 10.1186/s13062-023-00381-x (PMC10268499; doi:10.1186/s13062-023-00381-x)
Supplement: Supplementary file 4 — Supplementary Material 4 [file 13062_2023_381_MOESM4_ESM.docx]

Detailed Methods

**Study design.** All animal procedures used in this study were approved by the Animal Care and Use Committee of the Institute of Animal Sciences of Chinese Academy of Agricultural Sciences (IAS2018-1007). Mice were maintained in specific pathogen-free (SPF) environment under a light: dark cycle of 12:12 h, at a temperature of 23 ℃ and humidity of 50%–70%; they had free access to food (chow diet) and water [20-22].

*Animal Experiment I: NH_3_/H_2_S study* [20,22]**.** Three-week-old ICR male mice were dosed with phosphate buffered saline (PBS) as vehicle control (Control group) or with Na_2_S-50 mg/kg body weight (BW) + NH_4_Cl-50 mg/kg BW (NH_3_/H_2_S group) [23,24] once daily for 5 weeks. There were 60 mice/group. The volume of gavage was 0.1 ml/mouse/day. Subsequently, 30 mice/treatment were humanely terminated for the analysis of sperm quality and other parameters. A further 30 mice/treatment were mated with normal (untreated) ICR female mice (male: female; 1:2). After birth of the F1 litter, the number of live pups/litter was counted and all mice were raised similarly without further treatment. At the age of 8 weeks (F1), 30 male mice/treatment were humanely terminated for analysis of sperm quality and other parameters. A further 30 male mice/treatment were mated with normal ICR female mice (male: female; 1:2) and subsequently underwent a similar procedure. After birth of the F2 litter, the number of live pups/litter was counted and all mice were raised in a similar manner without further treatment (Study scheme in Fig. 1a).

*Animal Experiment II: Knockdown or overexpression of lncRNA5251 in mouse testes* [25,26]. The procedure for the production of shRNA and *In vivo* virus grafting have been published in our recent article [25]. (1) *Production of lentivirus.* Lentivirus production was performed as described previously [25,26]. Knockdown Lenti-*lncRNA5251* was cloned using the lentivirus-*3* (LV3) vector as a backbone (Supplementary Fig. 1a), while overexpression of lenti-lncRNA5251 was cloned using the lntivirus-5 (LV5) vector as a backbone (Supplementary Fig. 1b). There were three knockdown shRNAs at two different positions for lncRNA5251, and the sequences for each position and NC are listed in Supplementary Table 1. The full length of lncRNA5251 (Supplementary Table 2) was inserted into LV5 to make the overexpression lentivirus (NC was the LV5 vector). The efficiency and specificity of shRNA knockdown were determined by transfecting into 293T cells using Lipofectamine 2000 (Invitrogen, Waltham, MA, USA; #11668-027), followed by analysis at 60 h post-transfection by qPCR. Lentivirus production was then performed as shown in Supplementary Fig. 1c. Approximately 10^9^ infectious viral particles/ml were obtained. (2) *In vivo* virus grafting and sample collection. *In vivo* virus grafting was performed as previously described [25,26]. In current investigation, four-week-old ICR male mice were used. Briefly, four-week-old ICR male mice were anesthetized with isofluorane. Microinjections were performed using 26-gauge needles connected to a 100 μL syringe. Virus (3 μl with titer greater than 3×10^8^/ml) for each position for knockdown shRNA [in total 6 μl with a titer >6×10^8^/ml for 3NC, or lncRNA8276 (KD) individually] were mixed and then injected into the testes. For overexpression, virus (6 μl with titer greater than 6×10^8^/ml) for 5NC, or lncRNA8276 (OV) individually were mixed and then injected into the testes. Then the mice were raised regularly for five weeks till nine weeks of age (Fig. 2a; muF0). The mice mated with normal 8-week-age ICR female mice (male: female, 1:1) for four days. The male mice were kept for another four days, then terminated for collection samples and analysis. The female mice were maintained regularly till the delivery of offspring. The offspring was raised regularly till eight weeks age. Then the male offspring (muF1) were terminated for collection of samples and analysis (Fig. 2a).

*Cell culture experiment*: *Knockdown or overexpression of lncRNA8276 in mouse spermatogonia cell line C18-4 cells* [25]. The C18-4 cell line (mouse spermatogonia stem cells; Donated by Dr. Wenxian Zeng, Northwest A&F University) was held in DMEM/F12 (Gibco) supplemented with 10% (FBS), 2 mM L-glutamine (Invitrogen), and 100 U/ml penicillin and streptomycin (Invitrogen) [25,27,28]. The cells were transfected with shRNA in 6-well plates. For knockdown, two respective shRNAs for each position were mixed together (titer >3×10^8^/ml) with RNAi-mate for the transfection of C18-4, while for overexpression, shRNAs (titer >3×10^8^/ml) with RNAi-mate for the transfection of C18-4. The transfection medium was changed after 12 h. Stable transfected cells were cultured in a similar manner to the non-transfected cells in their respective media.

**Evaluation of spermatozoa motility using a computer-assisted sperm analysis system.** Spermatozoa motility was assessed using a computer-assisted sperm assay (CASA) method according to World Health Organization guidelines [20,21,29-31]. After euthanasia, spermatozoa were collected from the cauda epididymis of mice and suspended in DMEM/F12 medium with 10% FBS and incubated at 37.5 ℃ for 30 min; samples were then placed in a pre-warmed counting chamber. The micropic sperm class analyzer (CASA system) was used in this investigation. It was equipped with a 20-fold objective, a camera adaptor (Eclipse E200, Nikon, Japan), and a camera (acA780-75gc, Basler, Germany), and it was operated by an SCA sperm class analyzer (MICROPTIC S.L.). The classification of sperm motility was as follows: grade A linear velocity >22 μm s^-1^; grade B <22 μm s^-1^ and curvilinear velocity >5 μm s^-1^; grade C curvilinear velocity <5 μm s^-1^; and grade D = immotile spermatozoa. The spermatozoa motility data represented only grade A + grade B since only these two grades are considered to be functional.

**Morphological observations of spermatozoa.** The extracted murine caudal epididymides were placed in RPMI medium, finely chopped, and then Eosin Y (1%) was added for staining as described previously [20,21,31]. Spermatozoon abnormalities were then viewed using an optical microscope and were classified into head or tail morphological abnormalities: two heads, two tails, blunt hooks, and short tails. The examinations were repeated three times, and 500 spermatozoa per animal were scored.

**Assessment of acrosome integrity.** After harvest, mouse spermatozoa were incubated at 37.5 ℃ for 30 min, after which a drop of sperm suspension was uniformly smeared on a clean glass slide. Smeared slides were air dried and incubated in methanol for 2 min for fixation. After fixation, the slides were washed with PBS three times. Assessment of an intact acrosome was accomplished by staining the sperm with 0.025% Coomassie brilliant blue G-250 in 40% methanol for 20 min at room temperature (RT). The slides were then washed three times with PBS and mounted with 50% glycerol in PBS. Acrosomal integrity was determined by an intense staining on the anterior region of the sperm head under bright-field microscopy (AH3-RFCA, Olympus, Tokyo, Japan) and scored accordingly [20,21,31].

**RNA Isolation and RNA-seq analyses** [31]. Briefly, total RNA was isolated using TRIzol Reagent (Invitrogen) and purified using a Pure-Link1 RNA Mini Kit (Cat: 12183018A; Life Technologies) following the manufacturers’ protocol. Total RNA samples were first treated with DNase I to degrade any possible DNA contamination. Then the mRNA was enriched using oligo(dT) magnetic beads. Mixed with the fragmentation buffer, the mRNA was broken into short fragments (about 200 bp), after which, the first strand of cDNA was synthesized using a random hexamer-primer. Buffer, dNTPs, RNase H, and DNA polymerase I were added to synthesize the second strand. The double strand cDNA was purified with magnetic beads. Subsequently, 3'-end single nucleotide A (adenine) addition was performed. Finally, sequencing adaptors were ligated to the fragments. The fragments were enriched by PCR amplification. During the QC step, an Agilent 2100 Bioanaylzer and ABI StepOnePlus Real-Time PCR System were used to qualify and quantify the sample library. The library products were prepared for sequencing in an Illumina HiSeqTM 2500. The reads were mapped to reference genes using SOAPaligner (v. 2.20) with a maximum of two nucleotide mismatches allowed at the parameters of “-m 0 -x 1000 -s 40 -l 35 -v 3 -r 2”. The read number of each gene was transformed into RPKM (reads per kilo bases per million reads), and then differentially expressed genes were identified using the DEGseq package and the MARS (MA-plot-based method with random sampling model) method. The threshold was set as FDR ≤0.001 and an absolute value of log_2_ ratio ≥1 to judge the significance of the difference in gene expression. Then on the data were analyzed by GO enrichment, KEGG enrichment.

**Histopathological analysis.** Testicular tissues were fixed in 10% neutral buffered formalin, paraffin embedded, cut into 5 μm sections and subsequently stained with hematoxylin and eosin (H&E) for histopathological analysis [20,21,31].

**Western blotting.** Western blotting analysis of proteins was carried out as previously reported [20,21,31]. Briefly, testicular tissue or C18-4 cell samples were lysed in RIPA buffer containing the protease inhibitor cocktail from Sangong Biotech, Ltd. (Shanghai, China). Protein concentration was determined using a BCA kit (Beyotime Institute of Biotechnology, Shanghai, China). Goat anti-actin was used as a loading control. The information for primary antibodies (Abs) were listed in Supplementary Table 3. Secondary donkey anti-goat Ab (Cat no.: A0181) was purchased from Beyotime Institute of Biotechnology, and goat anti-rabbit (Cat no.: A24531) Abs were bought from Novex^®^ by Life Technologies (USA). Fifty micrograms of total protein per sample were loaded onto 10% SDS polyacrylamide electrophoresis gels. The gels were transferred to a polyvinylidene fluoride (PVDF) membrane at 300 mA for 2.5 h at 4 ℃. The membranes were then blocked with 5% bovine serum albumin (BSA) for 1 h at RT, followed by three washes with 0.1% Tween-20 in TBS (TBST). The membranes were incubated with primary Abs diluted with 1:500 in TBST with 1% BSA overnight at 4 ℃. After three washes with TBST, the blots were incubated with the HRP-labelled secondary goat anti-rabbit or donkey anti-goat Ab respectively for 1 h at RT. After three washes, the blots were imaged. The bands were quantified using Image-J software. The intensity of the specific protein band was normalized to actin first, then the data were normalized to the control. The experiment was repeated >6 times.

**Detection of protein levels and location in testis using immunofluorescence staining.** The methodology for immunofluorescence staining of testicular samples is reported in our recent publications [20,21,31]. Sections of testicular tissue (5 μm) were prepared and subjected to antigen retrieval and immunostaining as previously described. Briefly, sections were first blocked with normal goat serum in PBS, followed by incubation with primary Abs (Supplementary Table 3; 1:100 in PBS-0.5% Triton X-100; Bioss Co. Ltd. Beijing, PR China) at 4^o^C overnight. After a brief wash, sections were incubated with an Alexa 546-labeled goat anti-rabbit secondary Ab (1:100 in PBS; Molecular Probes, Eugene, OR, USA) at RT for 30 min and then counterstained with 4',6-diamidino-2-phenylindole (DAPI). The stained sections were examined using a Leica Laser Scanning Confocal Microscope (LEICA TCS SP5 II, Germany). Ten animal samples from each treatment group were analysed. Positively stained cells were counted. A minimum of 1000 cells were counted for each sample of each experiment. The data were then normalized to the control.

***Immunofluorescence staining with frozen sections for C18-4 cells*** [32]**.** The collected C18-4 cells from the different treatments were fixed with 4% PFA for 2 h at 4℃. Next, these cell pellets were incubated with agar gel for coating and then washed with PBS three times. The cell pellets were then incubated in 15% sucrose (in PBS) for 15 min and transferred to 30% sucrose (in PBS) for 15 min. Subsequently, the cell pellets were embedded in an optimal cutting temperature compound for frozen cell specimens. Further, the cell blocks were cut into 5 μm-thick sections using a freezing microtome and sections were washed in PBS three times. Sections were then blocked with 5% bovine serum albumin (BSA) for 30 min and incubated in primary antibodies for 2 h at 4℃ (Table S1). The sections were subsequently washed again with PBS and incubated for 1 h with the secondary antibody. Hoechst 33342 was used to stain cell nuclei. To measure the number of positive cells, the stained sections were visualized using a Nikon Eclipse TE2000‐U fluorescence microscope (Nikon, Inc) or confocal microscopy, and the captured fluorescent images were analyzed using ImagePro software.

**Statistical analysis.** Data were analyzed using SPSS statistical software (IBM Co., NY) with one-way analysis of variance (ANOVA) followed by LSD multiple comparison tests or T-test. The data were shown as the mean ± SEM. Statistical significance was based on p < 0.05.
