# Supplementary material for: LncRNA5251 inhibits spermatogenesis via modification of cell-cell junctions
Source: Biol Direct. 2023 Jun 15;18:31. doi: 10.1186/s13062-023-00381-x (PMC10268499; doi:10.1186/s13062-023-00381-x)
Supplement: Supplementary file 5 — Supplementary Material 5 [file 13062_2023_381_MOESM5_ESM.pdf]

```
            10        20        30        40        50        60
ACTTCGTATAGCATACATTATACGAAGTTATATTAAGGGTTCCAAGCTTAAGCGGCCGCGT
```

A1767_1-EF_1AF-Z170713122A_C08.ab1(50>600) →

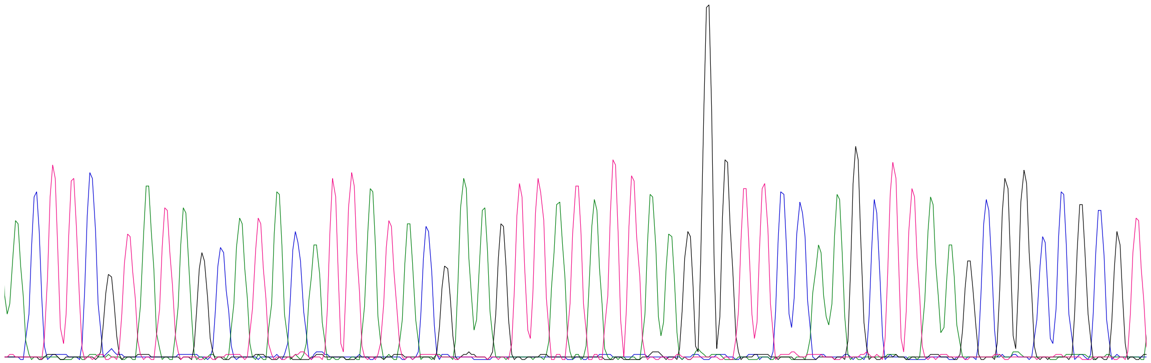

```
ACTTCGTATAGCATACATTATACGAAGTTATATTAAGGGTTCCAAGCTTAAGCGGCCGCGT
```

B3448 mlnc5251.seq(1>1571) →

```
                                                           GT
```

```
        70        80        90       100       110       120
AAATGGGTCTCAAGTTCTAGAGAGAAAACAGTAGTAGGCCTTGCTCAGTGTCCACCATGAA
```

A1767_1-EF_1AF-Z170713122A_C08.ab1(50>600) →

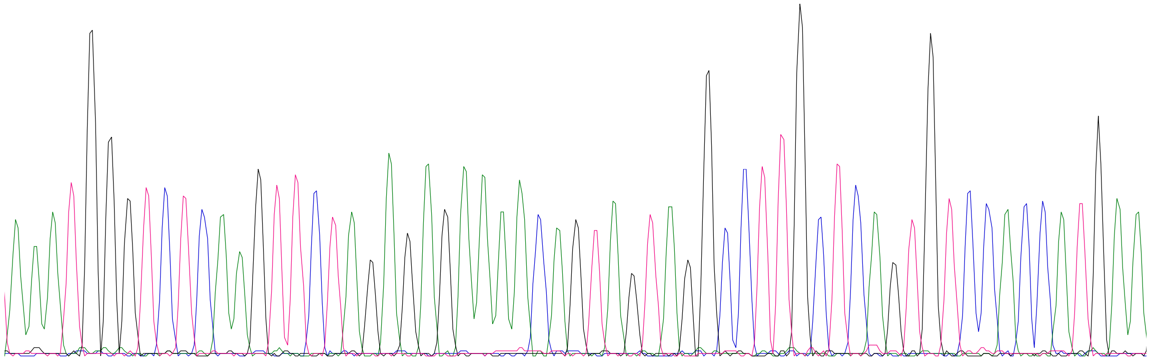

```
AAATGGGTCTCAAGTTCTAGAGAGAAAACAGTAGTAGGCCTTGCTCAGTGTCCACCATGAA
```

B3448 mlnc5251.seq(1>1571) →

```
AAATGGGTCTCAAGTTCTAGAGAGAAAACAGTAGTAGGCCTTGCTCAGTGTCCACCATGAA
```

```
       130       140       150       160       170       180
GTGCAGAAGGCACAGGTGTGACCTTTCCCCACCTTCAGGAACTCTTCACTGGCTAACGTTT
```

```
                                                        130       140       150       160       170       180
                                                   GTGCAGAAGGCACAGGTGTGACCTTTCCCCACCTTCAGGAACTCTTCACTGGCTAACGTTT
A1767_1-EF_1AF-Z170713122A_C08.ab1(50>600)    →
```

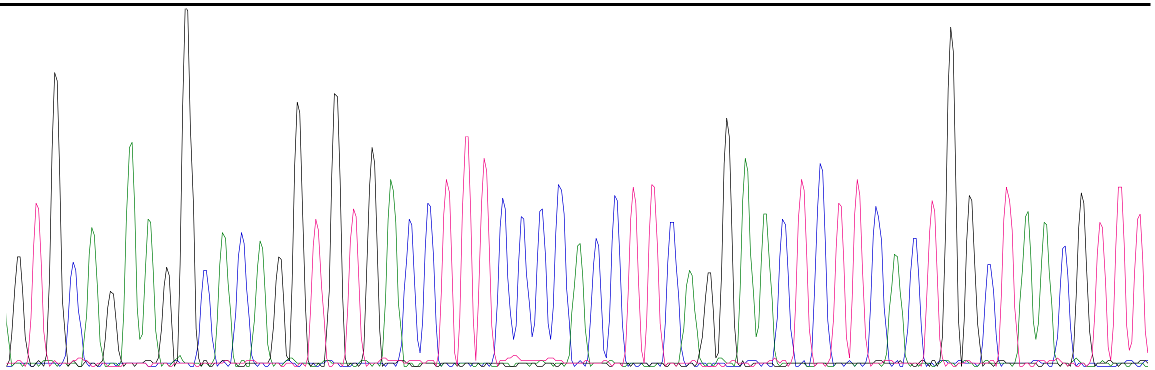

```
                                                   GTGCAGAAGGCACAGGTGTGACCTTTCCCCACCTTCAGGAACTCTTCACTGGCTAACGTTT
B3448 mlnc5251.seq(1>1571)                    →    GTGCAGAAGGCACAGGTGTGACCTTTCCCCACCTTCAGGAACTCTTCACTGGCTAACGTTT

                                                       190       200       210       220       230       240
                                                   TCAACACAATTTTTATTTCTTAAAGGCAAAATATTTTGTGTCCTGTGTTGCAAGATCACAA
A1767_1-EF_1AF-Z170713122A_C08.ab1(50>600)    →
```

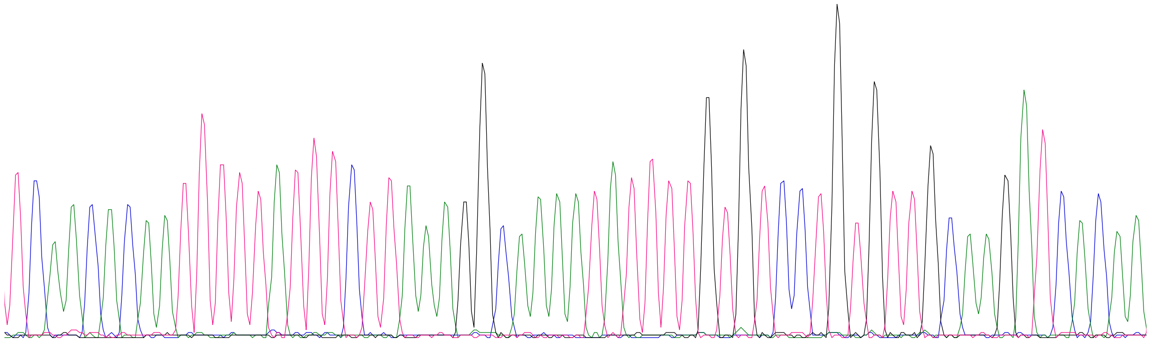

```
                                                   TCAACACAATTTTTATTTCTTAAAGGCAAAATATTTTGTGTCCTGTGTTGCAAGATCACAA
B3448 mlnc5251.seq(1>1571)                    →    TCAACACAATTTTTATTTCTTAAAGGCAAAATATTTTGTGTCCTGTGTTGCAAGATCACAA

                                                      250       260       270       280       290       300
                                                   TGGTTAGTAAAATCTTAGTATTTGGAAATAGAATAAGCTTGTTTCTTCCCTTTTAAAATGT
```

250 260 270 280 290 300

TGGTTAGTAAAATCTTAGTATTTGGAAATAGAATAAGCTTGTTTCTTCCCTTTTAAAATGT

A1767_1-EF_1AF-Z170713122A_C08.ab1(50>600) →

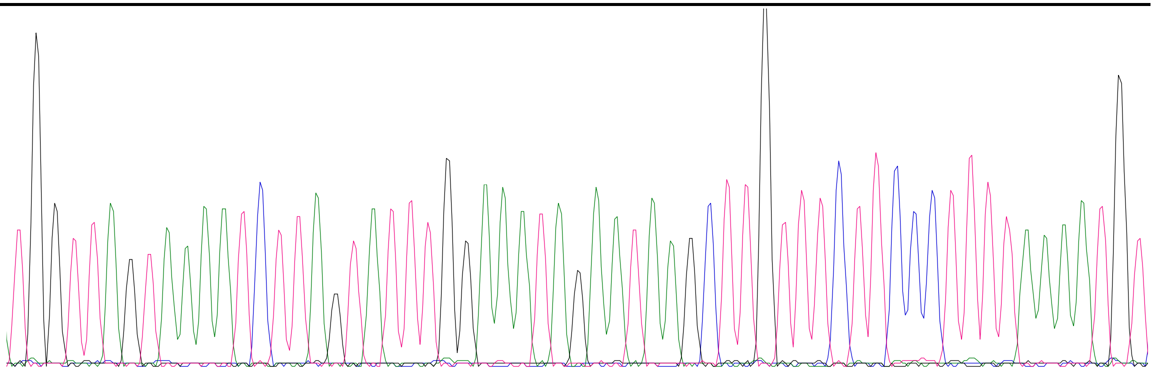

TGGTTAGTAAAATCTTAGTATTTGGAAATAGAATAAGCTTGTTTCTTCCCTTTTAAAATGT

B3448 mlnc5251.seq(1>1571) → TGGTTAGTAAAATCTTAGTATTTGGAAATAGAATAAGCTTGTTTCTTCCCTTTTAAAATGT

310 320 330 340 350 360

CTGTCTCAGAAATATAAGTCCAGGAAAGTCCAAGAAAATGAGCAGGGGCATACTCTGAGCA

A1767_1-EF_1AF-Z170713122A_C08.ab1(50>600) →

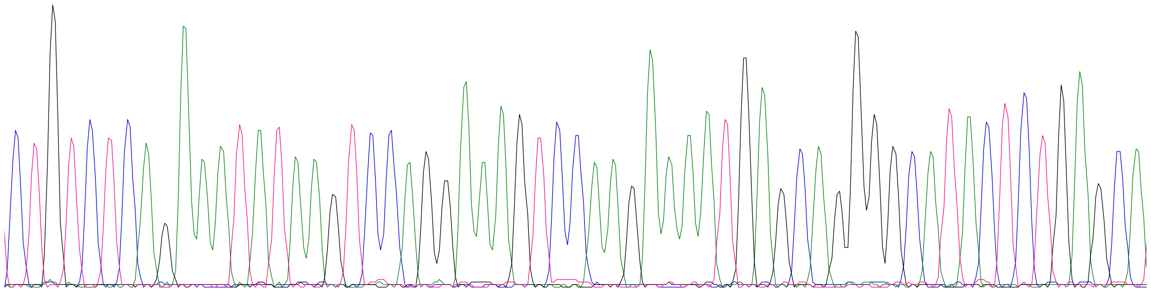

CTGTCTCAGAAATATAAGTCCAGGAAAGTCCAAGAAAATGAGCAGGGGCATACTCTGAGCA

B3448 mlnc5251.seq(1>1571) → CTGTCTCAGAAATATAAGTCCAGGAAAGTCCAAGAAAATGAGCAGGGGCATACTCTGAGCA

370 380 390 400 410 420

TGGTGTATGTGGGAATTTGGAGGTTTTGTAAAACCCTTTTCTCCAAGCCCATGTCACTATC

370 380 390 400 410 420

TGGTGTATGTGGGAATTTGGAGGTTTTGTAAAACCCTTTTCTCCAAGCCCATGTCACTATC

A1767_1-EF_1AF-Z170713122A_C08.ab1(50>600) →

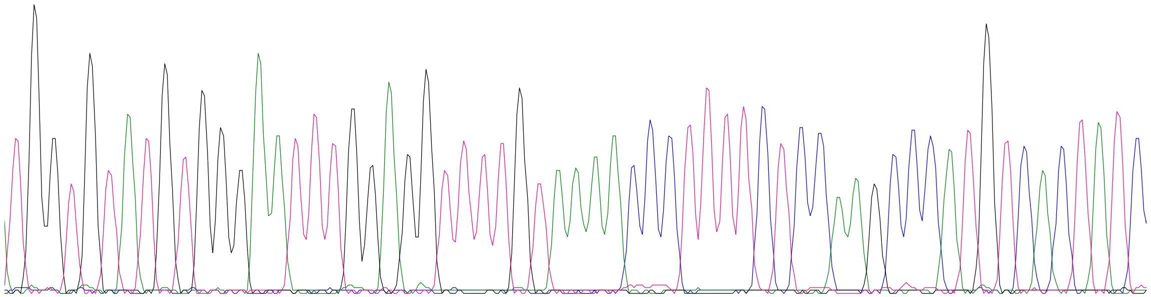

TGGTGTATGTGGGAATTTGGAGGTTTTGTAAAACCCTTTTCTCCAAGCCCATGTCACTATC

B3448 mlnc5251.seq(1>1571) → TGGTGTATGTGGGAATTTGGAGGTTTTGTAAAACCCTTTTCTCCAAGCCCATGTCACTATC

430 440 450 460 470 480

CATTGAACCAAAGTCCTCAGGAGTCTGGAAAGTCGGGAGGTTGGGCCAAAAGTCTAGAAAC

A1767_1-EF_1AF-Z170713122A_C08.ab1(50>600) →

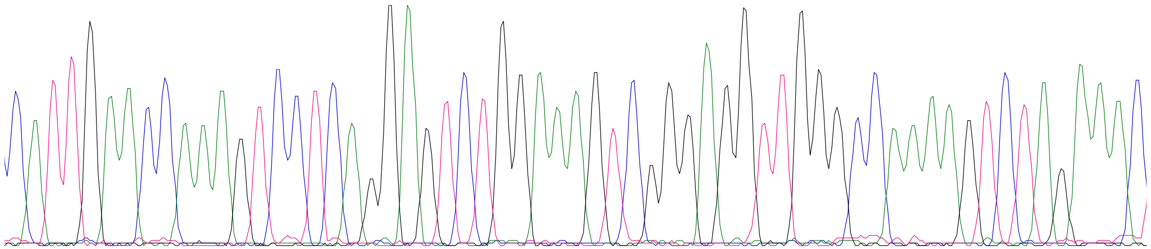

CATTGAACCAAAGTCCTCAGGAGTCTGGAAAGTCGGGAGGTTGGGCCAAAAGTCTAGAAAC

B3448 mlnc5251.seq(1>1571) → CATTGAACCAAAGTCCTCAGGAGTCTGGAAAGTCGGGAGGTTGGGCCAAAAGTCTAGAAAC

490 500 510 520 530 540

TTGGCTGTAGGTCAGCGTGACACACTTTTTTACAGAGTCTGTGGTTTTGCATATTTTTTTG

490 500 510 520 530 540

TTGGCTGTAGGTCAGCGTGACACACTTTTTTACAGAGTCTGTGGTTTTGCATATTTTTTTG

A1767_1-EF_1AF-Z170713122A_C08.ab1(50>600) →

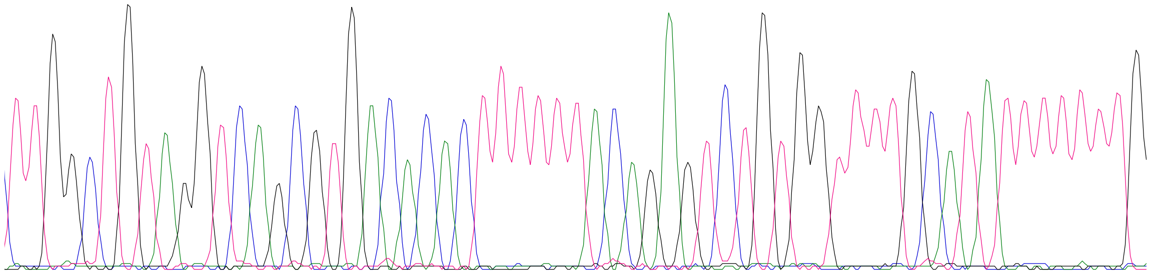

TTGGCTGTAGGTCAGCGTGACACACTTTTTTACAGAGTCTGTGGTTTTGCATATTTTTTTG

B3448 mlnc5251.seq(1>1571) → TTGGCTGTAGGTCAGCGTGACACACTTTTTTACAGAGTCTGTGGTTTTGCATATTTTTTTG

550 560 570 580 590 600 610

GCAGGCAAGTATATTTTAGTTATTTTTCTGTGACCTTGATATTTGAAATTAAGATTGTCAG

A1767_1-EF_1AF-Z170713122A_C08.ab1(50>600) →

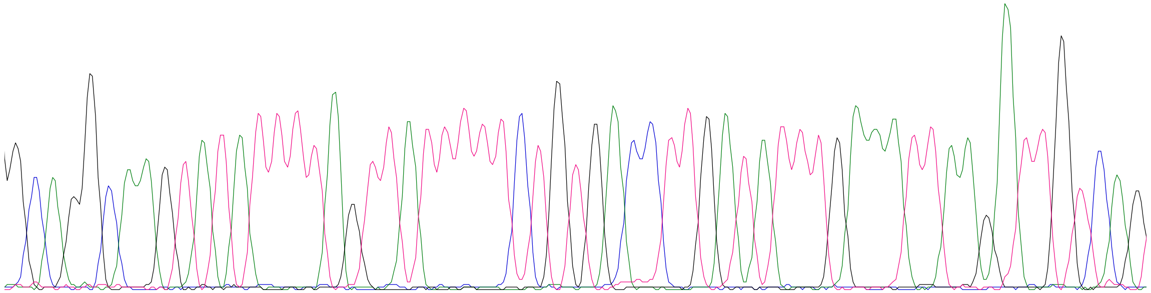

GCAGGCAAGTATATTTTAGTTATTTTTCTGTGACCTTGATATTTGAAATTAAGATTGTCAG

B3448 mlnc5251.seq(1>1571) → GCAGGCAAGTATATTTTAGTTATTTTTCTGTGACCTTGATATTTGAAATTAAGATTGTCAG

550 560 570 580 590 600 610

GCAGGCAAGTATATTTTAGTTATTTTTCTGTGACCTTGATATTTGAAATTAAGATTGTCAG

A1767_1-B3448F1-Z170713122A_E08.ab1(50>600) →

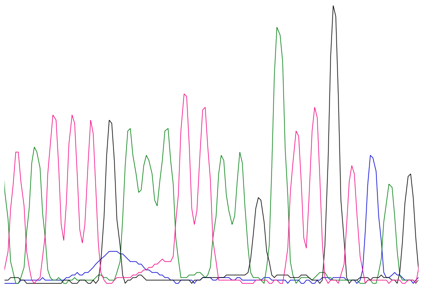

TATTTGAAATTAAGATTGTCAG

620 630 640 650 660 670

TGTATTTGGACTTTTTTTTTCCCCTGAAGTTAGAACAGTTGTATTAGGCATTTACTCTTGT

A1767_1-EF_1AF-Z170713122A_C08.ab1(50>600) →

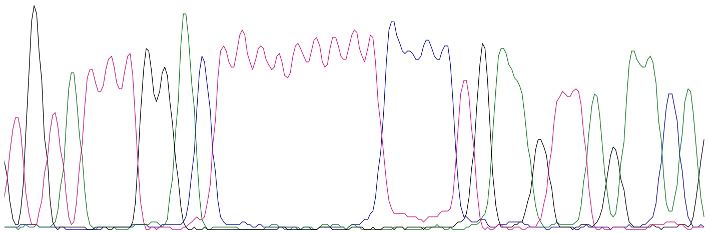

TGTATTTGGACTTTTTTTTTCCCCTGAAGTTAGAACA

B3448 mlnc5251.seq(1>1571) → TGTATTTGGACTTTTTTTTTCCCCTGAAGTTAGAACAGTTGTATTAGGCATTTACTCTTGT

A1767_1-B3448F1-Z170713122A_E08.ab1(50>600) →

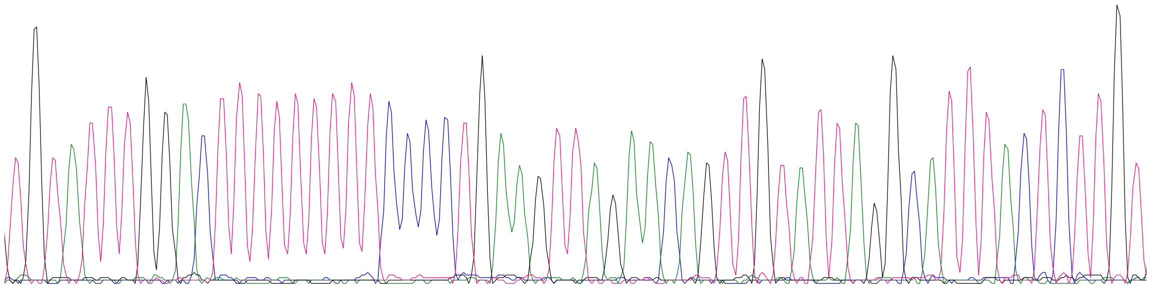

TGTATTTGGACTTTTTTTTTCCCCTGAAGTTAGAACAGTTGTATTAGGCATTTACTCTTGT

680 690 700 710 720 730

GAATCTTTATGCAGATGTCTTACCAGTCTTGTGAATAAAGTGCAGATGACATACTTTGTAT

B3448 mlnc5251.seq(1>1571) → GAATCTTTATGCAGATGTCTTACCAGTCTTGTGAATAAAGTGCAGATGACATACTTTGTAT
A1767_1-B3448F1-Z170713122A_E08.ab1(50>600) →

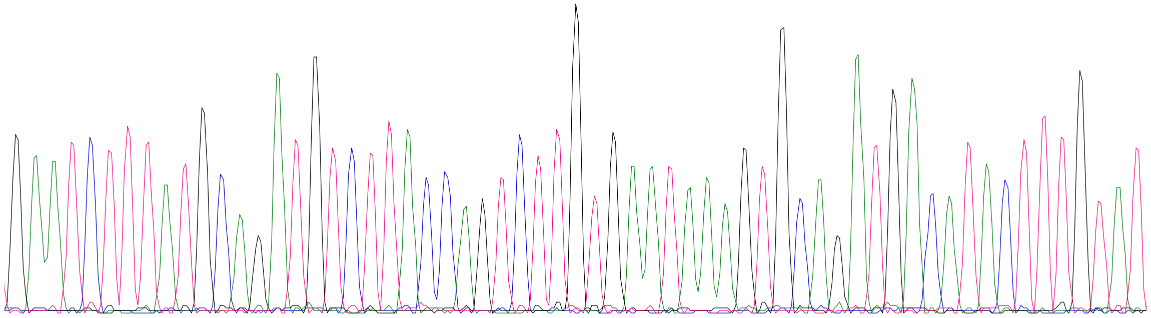

GAATCTTTATGCAGATGTCTTACCAGTCTTGTGAATAAAGTGCAGATGACATACTTTGTAT

740 750 760 770 780 790

TGTAGGAATCTCACTGCCCATCCTGTCTGCCAGTGGCTCTGTTAGGCTAGGTTCTTCACTC

B3448 mlnc5251.seq(1>1571) → TGTAGGAATCTCACTGCCCATCCTGTCTGCCAGTGGCTCTGTTAGGCTAGGTTCTTCACTC
A1767_1-B3448F1-Z170713122A_E08.ab1(50>600) →

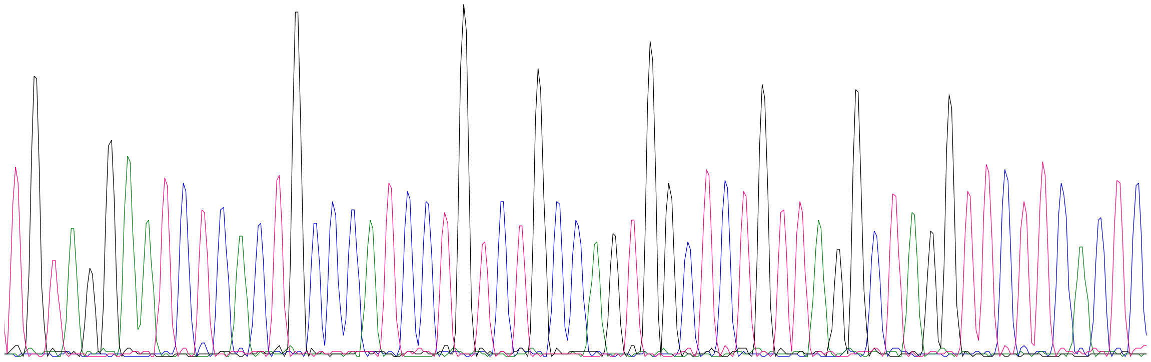

TGTAGGAATCTCACTGCCCATCCTGTCTGCCAGTGGCTCTGTTAGGCTAGGTTCTTCACTC

800 810 820 830 840 850

CAAAGCTGGGACTTAATGTCCTTCTAGTTGGGAATCTTCAGAAGTAATTTGAATGAGTTCA

B3448 mlnc5251.seq(1>1571) → CAAAGCTGGGACTTAATGTCCTTCTAGTTGGGAATCTTCAGAAGTAATTTGAATGAGTTCA
A1767_1-B3448F1-Z170713122A_E08.ab1(50>600) →

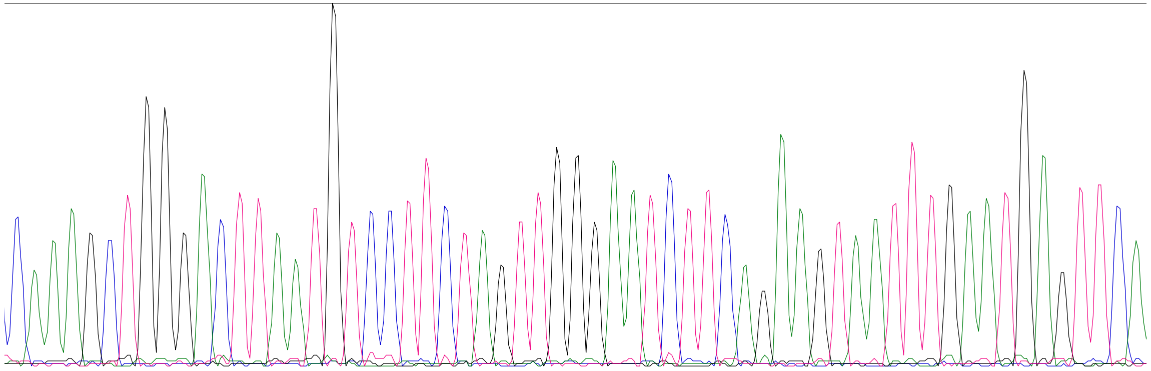

CAAAGCTGGGACTTAATGTCCTTCTAGTTGGGAATCTTCAGAAGTAATTTGAATGAGTTCA

860 870 880 890 900 910

AACCAAATCTTGATAGCAGGAGACAGCTTCCTGGTCTAGATGTACAATTAGCTTAGGTTGG

B3448 mlnc5251.seq(1>1571) → AACCAAATCTTGATAGCAGGAGACAGCTTCCTGGTCTAGATGTACAATTAGCTTAGGTTGG
A1767_1-B3448F1-Z170713122A_E08.ab1(50>600) →

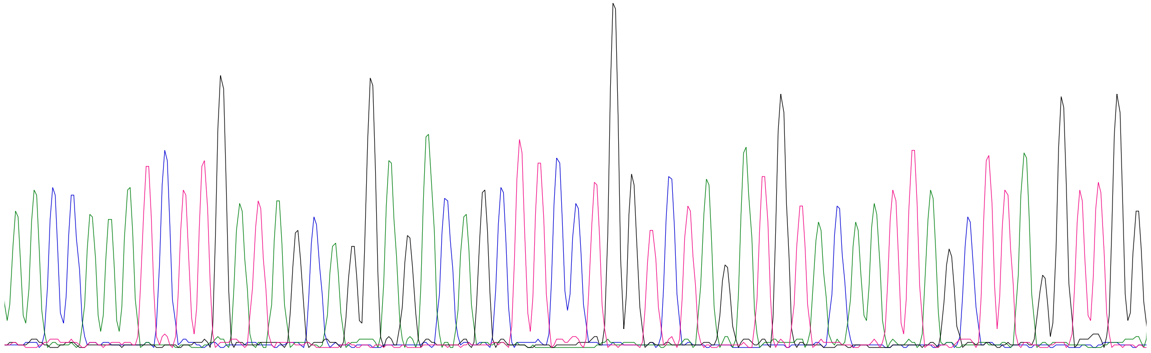

AACCAAATCTTGATAGCAGGAGACAGCTTCCTGGTCTAGATGTACAATTAGCTTAGGTTGG

```
                                                    920       930       940       950       960       970
                                                    AATTAAAGAGGGTTTGGGAATGTCTTTAGTCTCTGTGTAAATACCAACGTGCTTATTATGC
B3448 mlnc5251.seq(1>1571)                      →   AATTAAAGAGGGTTTGGGAATGTCTTTAGTCTCTGTGTAAATACCAACGTGCTTATTATGC
A1767_1-B3448F1-Z170713122A_E08.ab1(50>600)     →
```

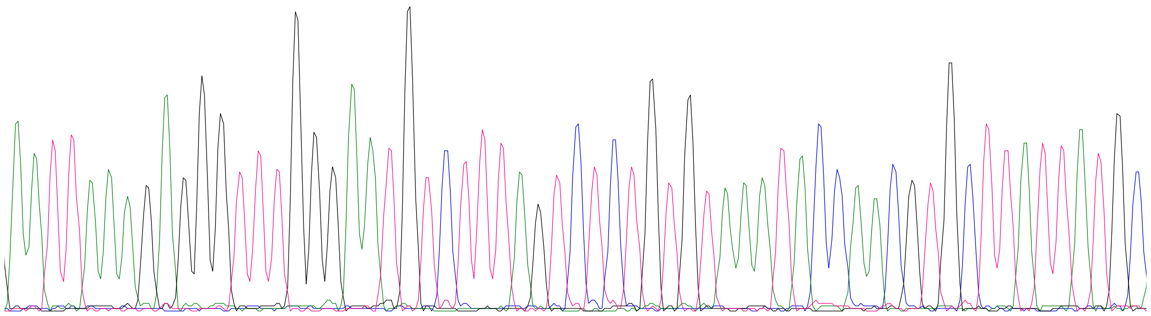

```
AATTAAAGAGGGTTTGGGAATGTCTTTAGTCTCTGTGTAAATACCAACGTGCTTATTATGC
```

```
                                                  980       990       1000      1010      1020      1030
                                                  ATCGTAAACCAGTGTGTATGCCTGTGTATGGGTCTGTAGAGCTGGTTTCTGCTTCAAGTGA
B3448 mlnc5251.seq(1>1571)                      →   ATCGTAAACCAGTGTGTATGCCTGTGTATGGGTCTGTAGAGCTGGTTTCTGCTTCAAGTGA
A1767_1-B3448F1-Z170713122A_E08.ab1(50>600)     →
```

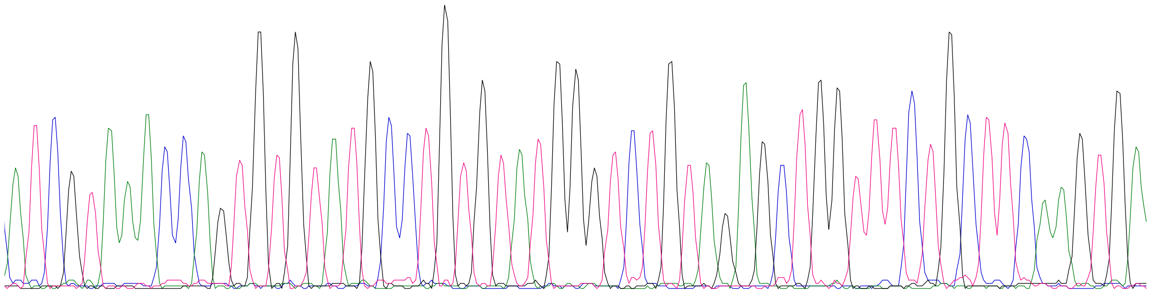

```
ATCGTAAACCAGTGTGTATGCCTGTGTATGGGTCTGTAGAGCTGGTTTCTGCTTCAAGTGA
```

1040 1050 1060 1070 1080 1090

AGCTGCACCTTTGATTTTATAAGGTCCCCTCCACCCGGAACCCTATAAACGTTTGTAAATA

B3448 mlnc5251.seq(1>1571) → AGCTGCACCTTTGATTTTATAAGGTCCCCTCCACCCGGAACCCTATAAACGTTTGTAAATA

A1767_1-B3448F1-Z170713122A_E08.ab1(50>600) →

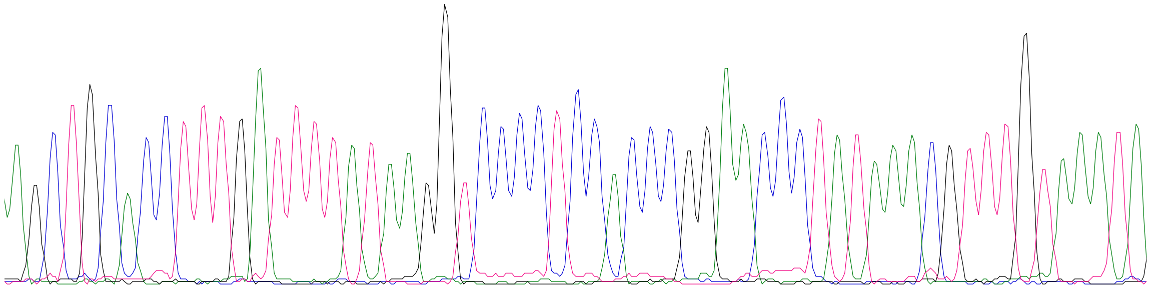

AGCTGCACCTTTGATTTTATAAGGTCCCCTCCACCCGGAACCCTATAAACGTTTGTAAATA

A1767_1-PIRES2_EGFP.P3-Z170713122A_D08.ab1(50>600) ←

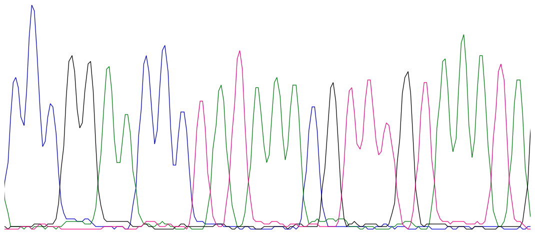

CCCGGAACCCTATAAACGTTTGTAAATA

1100 1110 1120 1130 1140 1150

GAACACTAAAATTTGTAGCGATAGGATCAATTTGGGAAATATCTGCTGAGAGACCAAAAAG

B3448 mlnc5251.seq(1>1571) → GAACACTAAAATTTGTAGCGATAGGATCAATTTGGGAAATATCTGCTGAGAGACCAAAAAG

A1767_1-B3448F1-Z170713122A_E08.ab1(50>600) →

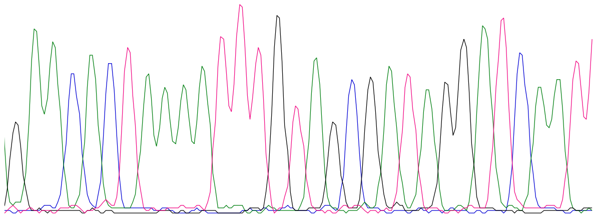

GAACACTAAAATTTGTAGCGATAGGATCAAT

A1767_1-PIRES2_EGFP.P3-Z170713122A_D08.ab1(50>600) ←

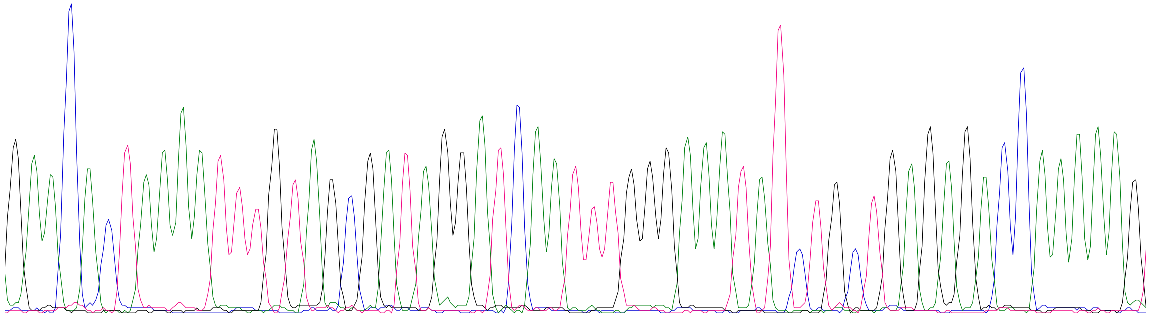

GAACACTAAAATTTGTAGCGATAGGATCAATTTGGGAAATATCTGCTGAGAGACCAAAAAG

```
                                              1160      1170      1180      1190      1200      1210      1220
                                              TTCATTTTTTTAAGTACCTTGGTTAAAGAGTAAAGATTATTCCTCTTATTTTTTAAAAGAA
B3448 mlnc5251.seq(1>1571)               →   TTCATTTTTTTAAGTACCTTGGTTAAAGAGTAAAGATTATTCCTCTTATTTTTTAAAAGAA
A1767_1-PIRES2_EGFP.P3-Z170713122A_D08.ab1(50>600) ←
```

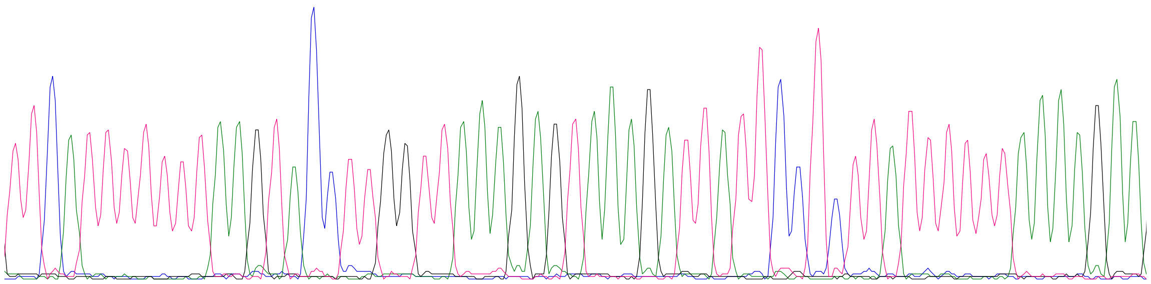

```
TTCATTTTTTTAAGTACCTTGGTTAAAGAGTAAAGATTATTCCTCTTATTTTTTAAAAGAA
```

```
                                              1230      1240      1250      1260      1270      1280
                                              GTATGCACTTTAACAAACACAGCTGCATGGGCAATTCAAACAAATCCATGAAGTGCAGTAC
B3448 mlnc5251.seq(1>1571)               →   GTATGCACTTTAACAAACACAGCTGCATGGGCAATTCAAACAAATCCATGAAGTGCAGTAC
A1767_1-PIRES2_EGFP.P3-Z170713122A_D08.ab1(50>600) ←
```

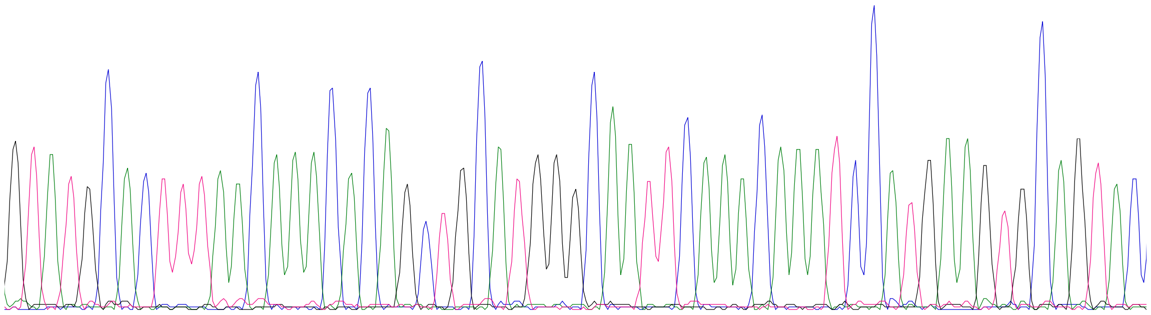

```
GTATGCACTTTAACAAACACAGCTGCATGGGCAATTCAAACAAATCCATGAAGTGCAGTAC
```

```
                                            1290      1300      1310      1320      1330      1340
                                            CCATCCAGAAACCACACTTCCTGAAAACCGTTCAAAGCAGAGTCCAGACGGGCTGGTGATC
B3448 mlnc5251.seq(1>1571)                →  CCATCCAGAAACCACACTTCCTGAAAACCGTTCAAAGCAGAGTCCAGACGGGCTGGTGATC
A1767_1-PIRES2_EGFP.P3-Z170713122A_D08.ab1(50>600) ←
```

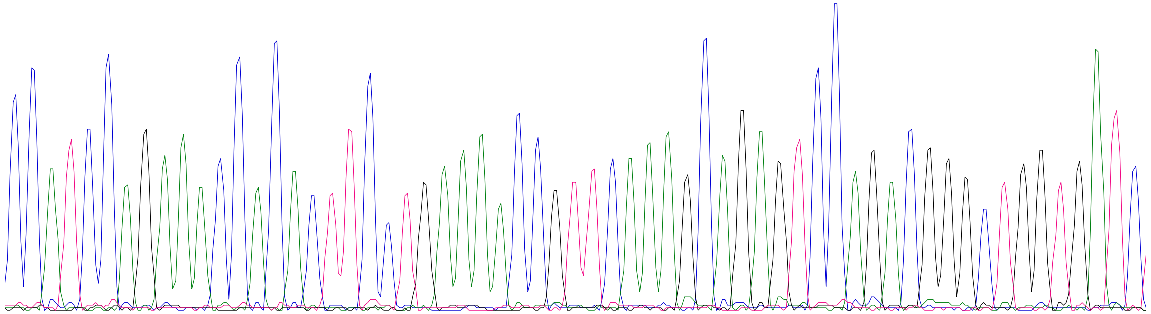

```
                                            CCATCCAGAAACCACACTTCCTGAAAACCGTTCAAAGCAGAGTCCAGACGGGCTGGTGATC
```

```
                                            1350      1360      1370      1380      1390      1400
                                            TCACTGCCTGTAGGTTGAAGCTCAGATTCTGATAAATTGTGAGACAAGCAGGGCTGCTTCA
B3448 mlnc5251.seq(1>1571)                →  TCACTGCCTGTAGGTTGAAGCTCAGATTCTGATAAATTGTGAGACAAGCAGGGCTGCTTCA
A1767_1-PIRES2_EGFP.P3-Z170713122A_D08.ab1(50>600) ←
```

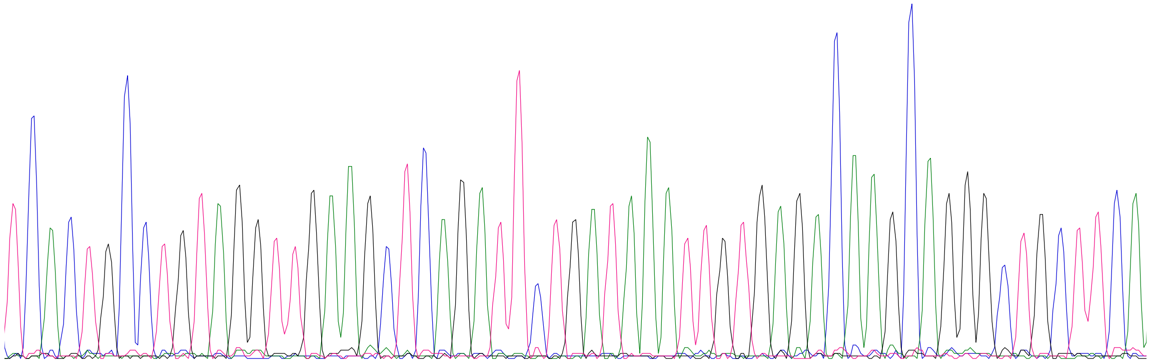

```
                                            TCACTGCCTGTAGGTTGAAGCTCAGATTCTGATAAATTGTGAGACAAGCAGGGCTGCTTCA
```

1410 1420 1430 1440 1450 1460

AAGAGCAATGTGAATACAGCCAGAAGCTTCAGACAGGTCTGTAAAATGGCGGGTCCCGTAT

B3448 mlnc5251.seq(1>1571) → AAGAGCAATGTGAATACAGCCAGAAGCTTCAGACAGGTCTGTAAAATGGCGGGTCCCGTAT

A1767_1-PIRES2_EGFP.P3-Z170713122A_D08.ab1(50>600) ←

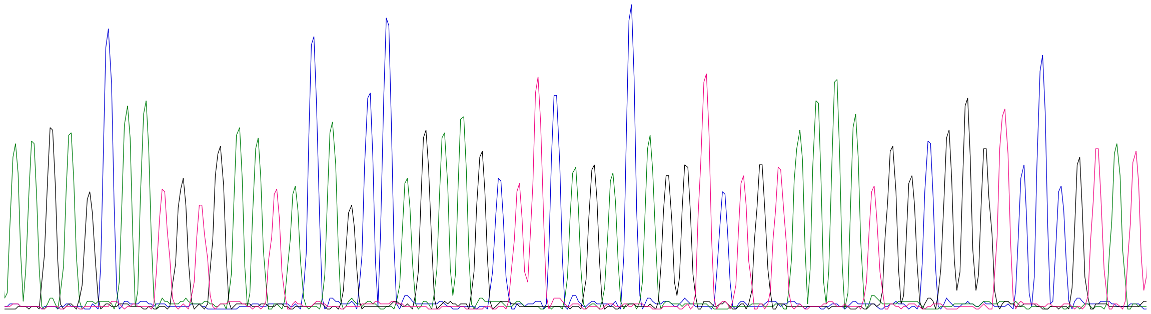

AAGAGCAATGTGAATACAGCCAGAAGCTTCAGACAGGTCTGTAAAATGGCGGGTCCCGTAT

1470 1480 1490 1500 1510 1520

TTACCACTAACTAGCAAAACTGACAGAAAAACTCATAGAAATAAAGTTAAGAATCCTTCCT

B3448 mlnc5251.seq(1>1571) → TTACCACTAACTAGCAAAACTGACAGAAAAACTCATAGAAATAAAGTTAAGAATCCTTCCT

A1767_1-PIRES2_EGFP.P3-Z170713122A_D08.ab1(50>600) ←

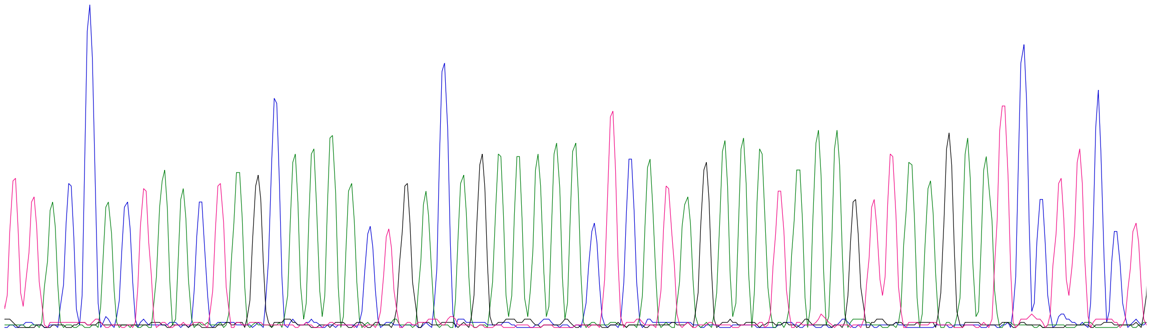

TTACCACTAACTAGCAAAACTGACAGAAAAACTCATAGAAATAAAGTTAAGAATCCTTCCT

1530 1540 1550 1560 1570 1580

GCTGGTGTGCACTCCTTCCAATAGCAGACTTTTGCAAATGGAGTTTTACAGTCTATATTTA

B3448 mlnc5251.seq(1>1571) → GCTGGTGTGCACTCCTTCCAATAGCAGACTTTTGCAAATGGAGTTTTACAGTCTATATTTA
A1767_1-PIRES2_EGFP.P3-Z170713122A_D08.ab1(50>600) ←

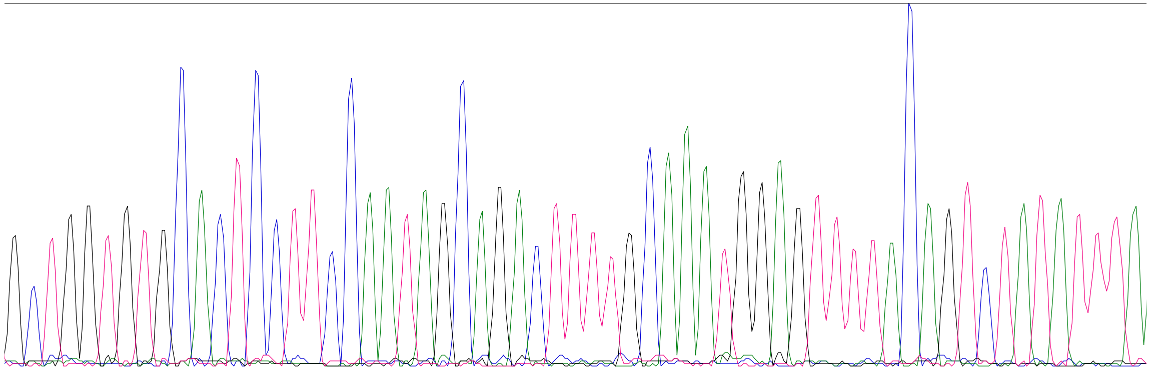

GCTGGTGTGCACTCCTTCCAATAGCAGACTTTTGCAAATGGAGTTTTACAGTCTATATTTA

1590 1600 1610 1620 1630 1640

AAAAATTGTATGTTTGTAACAAATAAAGTATGCGGAAAAGTGAAGGATCCGACACTCTCTA

B3448 mlnc5251.seq(1>1571) → AAAAATTGTATGTTTGTAACAAATAAAGTATGCGGAAAAGTGAA
A1767_1-PIRES2_EGFP.P3-Z170713122A_D08.ab1(50>600) ←

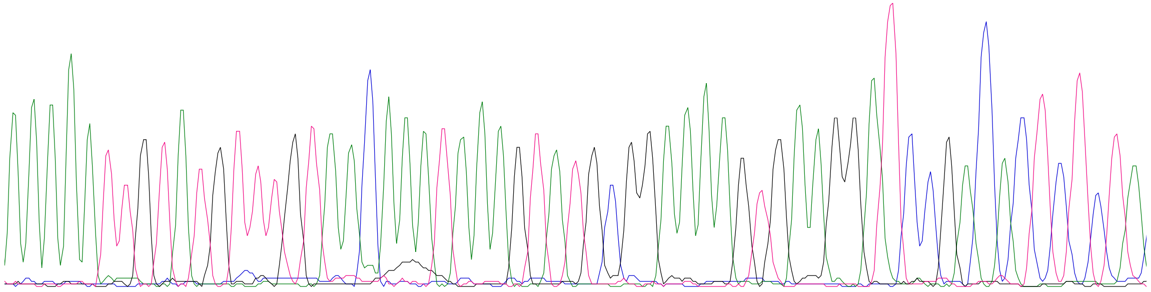

AAAAATTGTATGTTTGTAACAAATAAAGTATGCGGAAAAGTGAAGGATCCGACACTCTCTA

1650 1660 1670

CTGATACTCATCACTAACGTACT

1650 1660 1670

CTGATACTCATCACTAACGTACT

A1767_1-PIRES2_EGFP.P3-Z170713122A_D08.ab1(50>600) ←

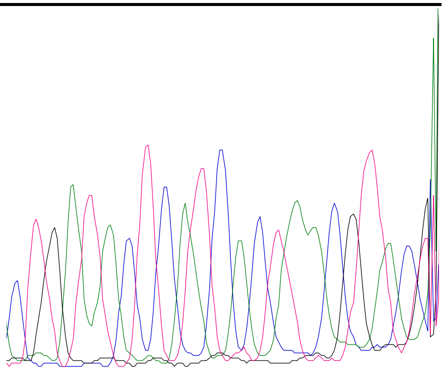

CTGATACTCATCACTAACGTACT
