# Supplementary material for: LncRNA5251 inhibits spermatogenesis via modification of cell-cell junctions
Source: Biol Direct. 2023 Jun 15;18:31. doi: 10.1186/s13062-023-00381-x (PMC10268499; doi:10.1186/s13062-023-00381-x)
Supplement: Supplementary file 6 — Supplementary Material 6 [file 13062_2023_381_MOESM6_ESM.docx]

**Additional information**

**Supplementary Fig. 1 Structure of the lentivirus vector used in this study and its production protocol for subsequent infection of cells and animals. a** Structure of LV3 vector. **b** Structure of LV5 vector. **c** Process of virus (shRNA).

**Supplementary Fig. 2 RAN-seq data for muF0 testis samples.** (**a**) Heatmap for RNA-seq data after knockdown lncRNA5251 in muF0 mouse testes. (**b**) Heatmap for RNA-seq data after overexpression lncRNA5251 in muF0 mouse testes. (**c**) GO enrichment analysis of down-regulated genes after inhibiting lncRNA5251 in muF0 mouse testes. (**d**) GO enrichment analysis of up-regulated genes after overexpressing lncRNA5251 in muF0 mouse testes.

**Supplementary Fig. 3.** **RAN-seq data for muF1 testis samples.** (**a**) Heatmap for RNA-seq data after knockdown lncRNA5251 in muF1 mouse testes. (**b**) Heatmap for RNA-seq data after overexpression lncRNA5251 in muF1 mouse testes. (**c**) GO enrichment analysis of down-regulated genes after inhibiting lncRNA5251 in muF1 mouse testes. (**d**) GO enrichment analysis of up-regulated genes after overexpressing lncRNA5251 in muF1 mouse testes.

**Supplementary Table 1.** Sequence for knockdown shRNA.

**Supplementary Table 2.** Sequence of lncRNA5251.

**Supplementary Table 3.** Primary antibody information.

**Supplementary file 1.** detailed methods.

**Supplementary file 2.** Alignment of lncRNA5251.
