# Supplementary material for: LncRNA5251 inhibits spermatogenesis via modification of cell-cell junctions
Source: Biol Direct. 2023 Jun 15;18:31. doi: 10.1186/s13062-023-00381-x (PMC10268499; doi:10.1186/s13062-023-00381-x)
Supplement: Supplementary file 7 — Supplementary Material 7 [file 13062_2023_381_MOESM7_ESM.docx]

**Supplementary Tables**

**Table S1 sequence for knockdown shRNA**

|  | sequence |
| --- | --- |
| Sh3NC (5’-3’) | TTCTCCGAACGTGTCACGT |
| shlncRNA5251 KD #1 (5’-3’) | GCTGGGACTTAATGTCCTTCT |
| ShlncRNA5251 KD #2 (5’-3’) | GCATGGGCAATTCAAACAAAT |
| ShlncRNA5251 KD #3 (5’-3’) | GCTTATTATGCATCGTAAACC |

**Table S2 sequence of lncRNA5251**

| **LncRNA5251 sequence** |
| --- |
| GTAAATGGGTCTCAAGTTCTAGAGAGAAAACAGTAGTAGGCCTTGCTCAGTGTCCACCATGAAGTGCAGAAGGCACAGGTGTGACCTTTCCCCACCTTCAGGAACTCTTCACTGGCTAACGTTTTCAACACAATTTTTATTTCTTAAAGGCAAAATATTTTGTGTCCTGTGTTGCAAGATCACAATGGTTAGTAAAATCTTAGTATTTGGAAATAGAATAAGCTTGTTTCTTCCCTTTTAAAATGTCTGTCTCAGAAATATAAGTCCAGGAAAGTCCAAGAAAATGAGCAGGGGCATACTCTGAGCATGGTGTATGTGGGAATTTGGAGGTTTTGTAAAACCCTTTTCTCCAAGCCCATGTCACTATCCATTGAACCAAAGTCCTCAGGAGTCTGGAAAGTCGGGAGGTTGGGCCAAAAGTCTAGAAACTTGGCTGTAGGTCAGCGTGACACACTTTTTTACAGAGTCTGTGGTTTTGCATATTTTTTTGGCAGGCAAGTATATTTTAGTTATTTTTCTGTGACCTTGATATTTGAAATTAAGATTGTCAGTGTATTTGGACTTTTTTTTTCCCCTGAAGTTAGAACAGTTGTATTAGGCATTTACTCTTGTGAATCTTTATGCAGATGTCTTACCAGTCTTGTGAATAAAGTGCAGATGACATACTTTGTATTGTAGGAATCTCACTGCCCATCCTGTCTGCCAGTGGCTCTGTTAGGCTAGGTTCTTCACTCCAAAGCTGGGACTTAATGTCCTTCTAGTTGGGAATCTTCAGAAGTAATTTGAATGAGTTCAAACCAAATCTTGATAGCAGGAGACAGCTTCCTGGTCTAGATGTACAATTAGCTTAGGTTGGAATTAAAGAGGGTTTGGGAATGTCTTTAGTCTCTGTGTAAATACCAACGTGCTTATTATGCATCGTAAACCAGTGTGTATGCCTGTGTATGGGTCTGTAGAGCTGGTTTCTGCTTCAAGTGAAGCTGCACCTTTGATTTTATAAGGTCCCCTCCACCCGGAACCCTATAAACGTTTGTAAATAGAACACTAAAATTTGTAGCGATAGGATCAATTTGGGAAATATCTGCTGAGAGACCAAAAAGTTCATTTTTTTAAGTACCTTGGTTAAAGAGTAAAGATTATTCCTCTTATTTTTTAAAAGAAGTATGCACTTTAACAAACACAGCTGCATGGGCAATTCAAACAAATCCATGAAGTGCAGTACCCATCCAGAAACCACACTTCCTGAAAACCGTTCAAAGCAGAGTCCAGACGGGCTGGTGATCTCACTGCCTGTAGGTTGAAGCTCAGATTCTGATAAATTGTGAGACAAGCAGGGCTGCTTCAAAGAGCAATGTGAATACAGCCAGAAGCTTCAGACAGGTCTGTAAAATGGCGGGTCCCGTATTTACCACTAACTAGCAAAACTGACAGAAAAACTCATAGAAATAAAGTTAAGAATCCTTCCTGCTGGTGTGCACTCCTTCCAATAGCAGACTTTTGCAAATGGAGTTTTACAGTCTATATTTAAAAAATTGTATGTTTGTAACAAATAAAGTATGCGGAAAAGTGAA |

Table S3 Primary antibody information

| **Gene symbol** | **Name** | **Cat. #** | **Predicted size** | **Source (Animal)** | **Company** |
| --- | --- | --- | --- | --- | --- |
| Actin | actin | Ab3280 | 42kDa | Rabbit (polyclonal) | Abcam |
| DDX4 (VASA) | DEAD (Asp Glu Ala Asp) box polypeptide | ab13840 | 76kDa | Rabbit (polyclonal) | Abcam |
| ODF1 | Outer defense fiber 1 | Sc-390152 | 27kd | Mouse (monoclonal) | Santa Cruz Biotechnology, Inc. |
| VCAM1 | Vascular Cell Adhesion Molecule 1 | bs-0920R | 81kDa | Rabbit (polyclonal) | Beijing Biosynthesis Biotechnology CO. |
| CADM2 | Cell Adhesion Molecule 2 | bs-8246R | 45kDa | Rabbit (polyclonal) | Beijing Biosynthesis Biotechnology CO. |
| TNP1(TP1) | Transition protein-1 | ab73135 | 10kDa | Rabbit (polyclonal) | Abcam |
| Cx37 | Connexin37 | bs-4067R | 37kDa | Rabbit (polyclonal) | Beijing Biosynthesis Biotechnology CO. |
| CYP11A1 | Cholesterol side chain cleavage enzyme | bs-10099R | 53/57kd | Rabbit (polyclonal) | Beijing Biosynthesis Biotechnology CO. |
| Bcl-xl | Bcl-xl | bs-1336R | 26kd | Rabbit (polyclonal) | Beijing Biosynthesis Biotechnology CO. |
| GDNF | Glial cell line derived neurotrophic factor | D220690 | 15kd | Rabbit (polyclonal) | Sangon Biotech (Shanghai) Co., Ltd. |
| Occludin | Rabbit Anti-Occludin antibody | bs-10011R | 59kDa | Rabbit (polyclonal) | Beijing Biosynthesis Biotechnology CO. |
| CX43 | Connexin 43 | bs-0651R | 42kDa | Rabbit (polyclonal) | Beijing Biosynthesis Biotechnology CO. |
| PGK2 | Anti-PGK2 rabbit polyclonal antibody | D121803 | 45kDa | Rabbit (polyclonal) | Sangon Biotech . |
| JAM1 | Junction adhesion molecule 1 | bs-3651R | 30kDa | Rabbit (polyclonal) | Beijing Biosynthesis Biotechnology CO. |
| Claudin 11 | Claudin 11 | bs-2183R | 22kd | Rabbit (polyclonal) | Beijing Biosynthesis Biotechnology CO. |
| DSG2 | Desmoglein 2 | ab150372 | 122kDa | Rabbit | Abcam |
| Catenin | Recombinant Anti-delta 1 Catenin/CAS antibody | Ab-92514 | 108kDa | Rabbit | Abcam |
| E-cadherin | Anti-E Cadherin | ab11512 | 125kDa | Rat(monoclonal) | Abcam |
| ZO-1 | ZO1 tight junction protein | Ab-190085 | 191kDa | Goat | Abcam |
|  |  |  |  |  |  |
